# Examining the e-cigarette scenario based on distribution, availability, marketing, and banning: A GIS-Based qualitative study in Bangladesh

**DOI:** 10.1371/journal.pone.0312802

**Published:** 2024-11-08

**Authors:** Md Jamil Hossain, Quazi Maksudur Rahman, Md. Abid Bin Siddique, Md Wahiduzzaman, Lakshmi Rani Kundu, Anika Bushra Boitchi, Ayesha Ahmed, Most. Zannatul Ferdous, Afifa Anjum, Md. Munir Mahmud, Md. Maruf Hasan, Tareq Mahmud, Md. Naim Pramanik, Meheruba Khan Sinthia, Tasmin Sayeed Nodi, Md. Mahadi Hassan, Soniya Akter Sony, Noushin Rahman Mahin, Md. Mosaraf Hossain, H. M. Miraz Mahmud, Md. Shakhaoat Hossain, Md. Tajuddin Sikder

**Affiliations:** 1 Department of Public Health and Informatics, Jahangirnagar University, Savar, Dhaka, Bangladesh; 2 Department of Psychiatry, University of Cambridge, Cambridge, United Kingdom; 3 Institute of Remote Sensing and GIS, Jahangirnagar University, Savar, Dhaka, Bangladesh; 4 Bangladesh Center for Communication Programs, Dhaka, Bangladesh; 5 Department of Social Relations, East West University, Dhaka, Bangladesh; AIIMS Jodhpur: All India Institute of Medical Sciences - Jodhpur, INDIA

## Abstract

**Background:**

Globally, over 81 million people use e-cigarettes, and the majority of them are young adults. Using e-cigarettes causes different types of adverse health effects both in adults and elderly people. Over time, using e-cigarettes has detrimental consequences on lung function, brain development and numerous other illnesses.

**Methods:**

This study employed a mixed-methods conducted between June and September 2023, comprising two phases: Geographical Information System (GIS) mapping of available e-cigarette point-of-sale (POS) locations and conducting 15 in-depth interviews (IDIs) with e-cigarette retailers, along with 5 key informant interviews (KIIs) involving tobacco control activists and policy experts. ArcGIS was employed for spatial analysis, creating distribution and type maps, and buffer and multi-buffer ring analyses were conducted to assess proximity to hospitals and academic institutions. Data analysis involved descriptive statistics for GIS mapping and qualitative analysis for interview transcripts, utilizing a priori codebook and thematic analysis.

**Results:**

A total of 276 POS were mapped in the entire Dhaka city. About 55 POS were found within 100m distance from academic institutions in Dhaka city, which offers the easy accessibility of young generations to e-cigarettes. The younger generation is becoming the major target for e-cigarettes because of their alluring flavors, appealing looks, and variation in flavors. Sellers have been using different marketing tactics such as postering, offering discounts and using internet marketing on social media. Moreover, they try to convince the customers by saying that e-cigarettes are ‘not harmful’ or ‘less harmful’. However, retailers were mostly taking e-cigarettes from local wholesalers or distributors. Customers buy these products both from in-store and online services. Due to the absence of laws and regulations on e-cigarettes in Bangladesh, the availability, marketing, and selling of e-cigarettes are increasing alarmingly.

**Conclusion:**

E-cigarette retail shops are mostly surrounded by academic institutions, and it is expanding. Besides, frequent exposure, easy accessibility, and tactful promotion encourage the younger generations to consume e-cigarettes. The government should take necessary control measures on manufacturing, storage, advertising, promotion, sponsorship, marketing, distribution, sale, import, and export in order to safeguard the health and safety of young and future generations.

## 1. Background

Electronic cigarettes, also known as e-cigarettes, are electronic devices that deliver nicotine in a vapor form to experience smoking traditional cigarettes [1]. These battery-powered devices operate by heating a mixture of propylene glycol, glycerin, and water with a variety of flavors [2]. From 2011 to 2018, there was a significant increase in the number of e-cigarette users, rising from approximately 7 million to 41 million. This shift made a great impact on consumption pattern and brought about a new era of tobacco consumption [3]. However, despite becoming increasingly popular, e-cigarettes pose serious threats to public health. Initially hailed as safer alternatives to conventional cigarettes, e-cigarettes introduce a novel source of nicotine, a highly addictive substance with so many adverse health effects [4–6]. Additionally, the World Health Organization has reported that e-cigarettes are harmful to health [7]. The heated liquid in the e-cigarette produces an aerosol of small particles that are absorbed by the lungs, quickly passing through the heart, and providing nicotine to the brain [8]. Even the regular use of e-cigarettes has been associated with deaths [9]. Notably, most e-cigarette users are former smokers or dual users, consuming traditional tobacco products, while a smaller group of people who have never smoked [10]. Although e-cigarettes have become increasingly popular among youth and young adults, but its consumption remained low among older adults. The jobless and manual laborers have the highest prevalence of e-cigarette usage, and men are more likely than women to use them [11]. It has found that about 700,000 middle and high school students are vaping every single day [12].Several research papers have depicted the reason why people are more likely to consume e-cigarettes. According to the Food and Drug Administration (FDA),teens who vape may become addicted to nicotine faster than teens who smoke cigarettes, leading to increased vaping frequency and duration, putting them at risk for nicotine addiction [13]. As a result, most of the customers (91%) reported enjoying vaping more than smoking, with 80% preferring non-tobacco e-cigarette flavors. In total, there were nearly 270 different e-cigarette brands available as of December 2022, an increase of 46.2% from January 2020 [14]. A more recent analysis found that these brands offer over 9,000 different devices, which has tripled since 2020 [15]. Researchers also identified more than 15,500 unique e-cigarette flavors available online in 2007 [16]. Among youth users of flavored e-cigarettes, the most commonly used flavor types were fruit (69.1%), candy/desserts/other sweets (38.3%), mint (29.4%) and menthol (26.6%) [12]. An experimental study found that adolescents who were exposed to displays predominantly comprising tobacco products were more willing to use e-cigarettes in the future, compared with those were not exposed to the displays [17]. Moreover, the effects of both e-cigarette retail display visibility, and the availability (both relative and absolute) of products such as food and alcohol and, by extension their visibility, is one of the key factor to change behaviors [18, 19]. According to a another study, the convincing pitches in advertisements greatly influence customer reactions to e-cigarette marketing and their behaviors and actions [20]. E-cigarette advertisements differ from traditional cigarette ads because they often portray their products as healthier alternatives to cigarettes, which can be used as solutions to help people quit smoking [20–23]. A review of various research articles shows that young people in developed countries like Europe and America are more attracted towards online exposure to e-cigarette retail displays on [24, 25]. According to a study, More than 40% of online e-cigarette vendors use loyalty programs, special discounts, and promotional coupons to attract new clients [26]. They also frequently utilize social media to distribute promotional deals, with an average of 2.6 different social media platforms used per seller website [26]. In the U.S., a large proportion of e-cigarettes in the market are imported, with nearly 100% coming from China between 2016 and 2018 [27]. In Australia, there are two possible ways to obtain an e-cigarette: the first is through a pharmacy, and the second is by using the Therapeutic Goods Administration (TGA) personal importation system to import up to three months’ worth of stock [28]. The legal status of e-cigarettes is currently pending in many countries [29]. Many countries such as Brazil, Singapore, Uruguay [30] and India have banned e-cigarettes [31]. The US Food and Drug Administration has issued some regulations for e-cigarettes, including supervision, a minimum buying age of 18 years, premarket approval applications, warning signs, and displaying the list of harmful ingredients [32]. The Government of Bangladesh (GoB) has now banned conventional smoking in specific public places and on public transport. There are also implemented some regulations for packaging and advertising of these tobacco products [33]. This law significantly reduces the prevalence of smoking and tobacco use among the population. In contrast, there has no laws and regulations regarding the prohibition of the use, selling, promotion, or advertising of e-cigarettes in Bangladesh [34, 35].

To achieve good health and wellbeing (SDG goal 3), we have to strengthen the effort on Tobacco Control by 2030 [36]. Bangladesh government has targeted to achieve tobacco free Bangladesh by 2040. As a developing country, However, Bangladesh currently lack of evidence regarding distribution, marketing, and policy initiatives of e-cigarettes. Additionally, the regulation of e-cigarettes in Dhaka and Bangladesh remains relatively underdeveloped, emphasizing a critical gap in the country’s approach to tobacco control. Thus, this study helps to bridge these gaps by exploring the perception, distribution, availability, planning and banning scenario of e-cigarettes in the retail market of Dhaka, the center of Bangladesh. The aim of this study to provide scientific evidence that can inform future regulatory efforts and enforcing law.

## 2. Methodology

### 2.1 Study design and setting

This was a qualitative study conducted between June and September 2023. Moreover, this study has been divided into two phases regarding data collection. The first phase generated data concerning Geographical Information System (GIS) mapping of the point of sale (POS) of e-cigarettes, and the second phase included in-depth interviews (IDIs) with e-cigarette retailers as well as key informant interviews (KIIs) with anti-tobacco activists and policymakers at both Dhaka North City Corporation (DNCC) and Dhaka South City Corporation (DSCC) in Bangladesh, which is also one of the world’s largest and most densely populated cities [37].

### 2.2 Study participants and sampling technique

In this study, the participants for in-depth interviews (IDIs) were such as e-cigarette vendors who were directly or indirectly involved in e-cigarette distribution, and the participants for the key informant interview (KII) were such as anti-tobacco activists of Dhaka city. Most of the literature advises measuring purposeful sample sizes inductively and keeping sampling until saturation. A total of 20 people were invited to participate in the interview including fifteen participants for IDI and five anti-tobacco activists for KII. However, we got our saturation level before finishing the 20 interviews. The inclusion criteria of the participants were being Bangladeshi citizens and residents, handling e-cigarettes, and ability to talk. On the contrary, the exclusion criteria were including not living in Bangladesh, being a resident without Bangladeshi, do not handling e-cigarette, not willing to participate in the study. Before each of the interviews, the participants were informed about the aim and objectives of the study, if they gave permission then interviews were conducted by the moderators. This study used purposive sampling techniques to the interviewees. The current study purposefully collected data from various participant settings (e.g., street e-cigarette shops, residential e-cigarette shops, hybrid e-cigarette shops, dedicated e-cigarette shops etc.) to ensure the strength of the data.

### 2.3 Data collection tools and techniques

#### 2.3.1 Geographical mapping

For GIS mapping, all e-cigarette outlets in the study area and their GPS coordinates were recorded. Stores that sold any kind of e-cigarette were considered as point of sales (POS). E-cigarette outlet coordinates were collected by six pairs of trained enumerators after attending 3 days of training session. The enumerators determined the e-cigarette outlet by looking for e-cigarette displays, seeing the purchase of e-cigarettes, or approaching the retailer personally in cases when none of these were apparent. The enumerators completed an electronic checklist on Kobo Toolbox embedded in their mobile phone. It included POS ID, name of POS, type of POS, GPS coordinates of e-cigarette shops and selling points, advertisements placed in the shop, and any institutions located near 100m and 500m within the shop. In this section, this study analyzed the distribution of e-cigarette shops, and types of shops, and assessed their proximity to academic institutions and hospitals. The collected GPS points are extracted from Kobo Toolbox and imported into GIS software, specifically ArcGIS for spatial analysis. For GIS mapping, this study used ArcGIS Pro software by ESRI (licensed version) and the scale is 1:100000. The POS location points collected from fields are plotted in the GIS attribute for mapping and showing the distribution of those POS. All the GIS maps are solely developed by the author. The output maps are produced using projected coordinate system of UTM (Zone 45N). In the GIS environment, firstly a distribution map of e-cigarette shops across Dhaka City was formed, encompassing both the North and South City Corporation areas. This map visually represents the spatial dispersion of e-cigarette shops. Additionally, a secondary map is generated to categorize the several types of e-cigarette shops. This provides essential information on the specific classifications or categories of e-cigarette shops present within the study area. Moving forward, a buffer analysis is undertaken to ascertain the distribution of e-cigarette shops within a defined radius of hospitals. In this case, a buffer zone of 100 meters is established around hospital locations to identify which e-cigarette shops fall within this designated zone. To further assess the proximity of academic institutions to e-cigarette shops, a multi-buffer ring analysis was conducted to determine the presence of academic institutions within specified distances, such as 100 meters and 500 meters, from e-cigarette shops. Multiple buffer zones were created around the e-cigarette shop locations to facilitate this evaluation.

#### 2.3.2 Conducting IDI and KIIs

The qualitative interview guideline was formed through the analysis of pertinent and significant literature, and pre-testing was carried out before of the data collection. Through the use of an open-ended questionnaire, data were gathered based on the respondents’ convenience. Before of the interview, brief descriptions of the study’s background, objectives, eligibility requirements, confidentiality, anonymity, and informed consent of each responder were provided and explained pleasantly to the interviewees and attached also in the first page of the guideline. The IDI guideline was formed, including questions such as perceptions regarding e-cigarettes, the e-cigarette distribution process, marketing strategies concerning e-cigarette distribution, and so on. In addition, the KII guideline was also formed based on perceptions about e-cigarettes, the import mechanism of e-cigarettes and how to reduce the importation of e-cigarettes, the concept of distribution and marketing strategy of e-cigarettes, if there is any support for reducing/banning e-cigarette use in Bangladesh, and so on. Prior to the interview, each respondent was provided full informed consent and made sure that the participants understood the reasons for inviting, the risks, privacy, anonymity, and confidentiality, as well as their right to withdraw and not participate. In the interview, we also ensured that they were under no obligation to continue and might end at any time. Interviewers made an effort to find a location with the fewest disruptions. Most of the interviews were around 45 min, and the audio was recorded to ensure validity. Additionally, we got their permission to record the interview on audio. The field notes were also taken to address any extra essential information. To facilitate the exploration of the inner meaning and uncover the phenomenon under investigation, data were gathered in the native language of the respondents. To maintain the anonymity of each respondent, we assigned ID numbers to them. The interviews had to be transcribed verbatim, coded, and summarized by the proficient native Bengali data enumerators. Finally, no incentives were provided to study participants to encourage their participation.

### 2.4 Data management and analysis

This study created geographical maps with ArcGIS Pro software by ESRI. Descriptive statistics (frequency and percentage) were computed to find out the proportion of POS in each city corporation and the percentage of POS types. On the other hand, transcripts of the interviews were read and reviewed carefully, coded under different themes, and finally were analyzed by narrative review method. To ensure accuracy, the transcripts and audio recordings were cross-checked for any inconsistencies. A priori codebook was used, which was developed through reviewing relevant literature and the thematic analysis was performed following the themes identifying techniques [38], where texts were extracted and summarized through manual coding. Finally, to explain the results, the data were methodically indexed, synthesized, and evaluated.

### 2.5 Ethical standard

The interviews were conducted in accordance with the 1964 Helsinki Declaration, and the highest ethical standards were maintained throughout the study. Ethical permission was obtained from the institutional review board (IRB), ‘Biosafety, Biosecurity & Ethical Committee’ (Ref No: BBEC, JU/M 2023/05 (30)) of the Jahangirnagar University, Savar, Dhaka. Furthermore, individual informed consent was taken from all participants who read/understood the purpose of the study before data collection.

## 3. Results

### 3.1 Baseline scenario of e-cigarette in Dhaka City

We found a total of 276 e-cigarette shops in Dhaka City, among them the majority (56.5%) were in Dhaka North City Corporation followed by Dhaka South City Corporation (43.5%). Among the POS in both South and North city corporations, the highest POS type was watch or sunglass shops (26.8%) followed by dedicated vape shops (26.4%), cigarette shops (17.4%), and cosmetics shops (12.7%), electronics shops (4%) etc. (Table 1).

**Table 1:** Frequency distribution of e-cigarette shops and POS types in Dhaka city.

Location	Frequency (n)	Percentage (%)
Dhaka North City Corporation	156	56.5
Dhaka South City Corporation	120	43.5
Total	276	100.0
**Types of POS**		
Watch/sunglass	74	26.8
Dedicated	73	26.4
Cigarette	48	17.4
Cosmetics	35	12.7
Electronics	11	4.0
Confectionary	7	2.5
Leather shop	5	1.8
Lighter and Refill	5	1.8
Mobile shop	3	1.1
Super shop	2	0.7
Tea	2	0.7
Others	9	4.1
**Total**	**276**	**100.0**

**Fig 1:**
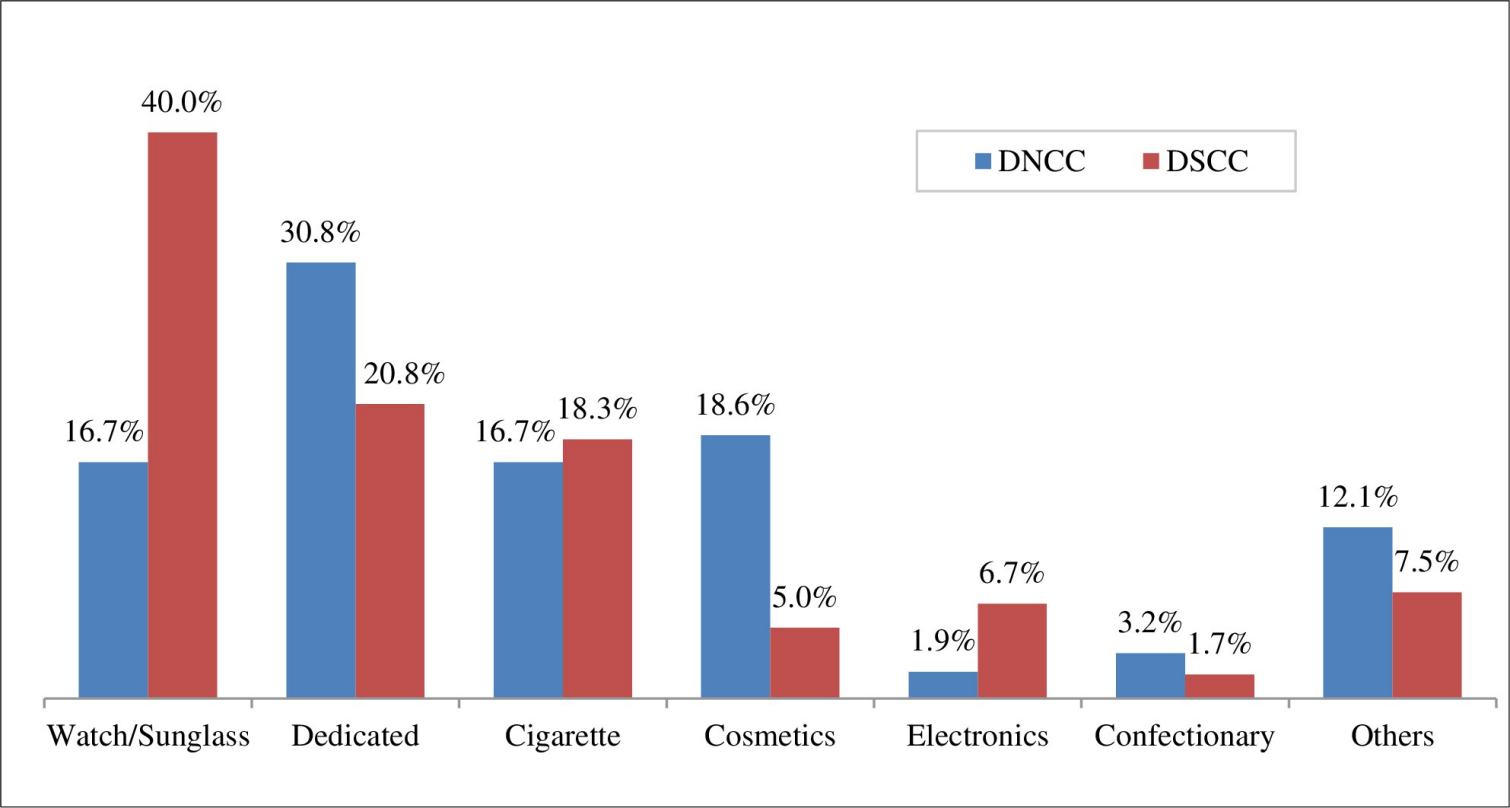
Distribution of POS types by city corporation. The bar graph illustrates the percentage of POS distribution based on City Corporation. In DSCC, the highest percentage POS type was watches and sunglasses shops (40%) followed by dedicated vape shops (20.8%), cigarette shops (18.3%). On the other hand, in DNCC the majority was dedicated vape shops (30.8%), while the same proportion of watch/sunglass and cigarettes shops were noticed at 16.7%.

The bar graph illustrates the percentage of POS distribution based on City Corporation. In DSCC, the highest percentage POS type was watches and sunglasses shops (40%) followed by dedicated vape shops (20.8%), cigarette shops (18.3%). On the other hand, in DNCC majority was dedicated vape shops (30.8%), while the same proportion of watch/sunglass and cigarettes shops were noticed at 16.7% (Fig 1). The distribution of several institutions is presented in Table 2.

**Table 2:** Institutions within 100 meters from the POS.

Type of Institutions*	Frequency (*n*)	Percentage
College	39	48.1
High School	36	44.4
Hospital	15	18.5
Primary School	13	16.0
Bank	13	16.0
Coaching center	12	14.8
Clinic	9	11.1
University	8	9.9
Kindergarten	1	1.2
***Base*, *n***	***276***	

*Multiple responses

**Fig 2:**
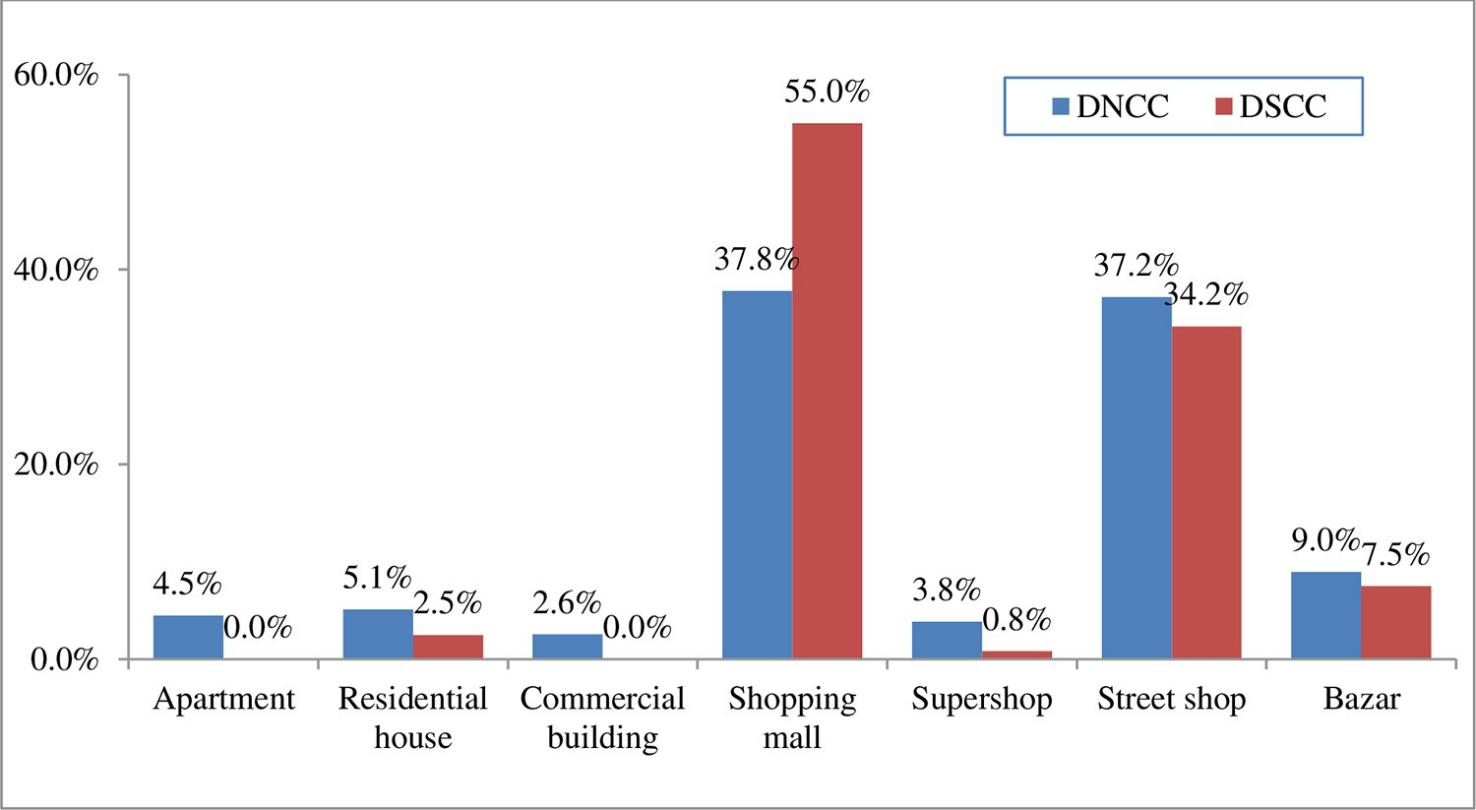
POS Location based on city corporation. The distribution of the location of POS based on City Corporation is depicted. It is observed that most of the POS located at shopping malls in both city corporations, whereas 55.0% in DSCC and 37.8% in DNCC followed by 5.1% (DNCC) and 2.5% (DSCC) in residential house, 3.8% (DNCC) and 0.8% (DSCC) in super shop.

**Fig 3:**
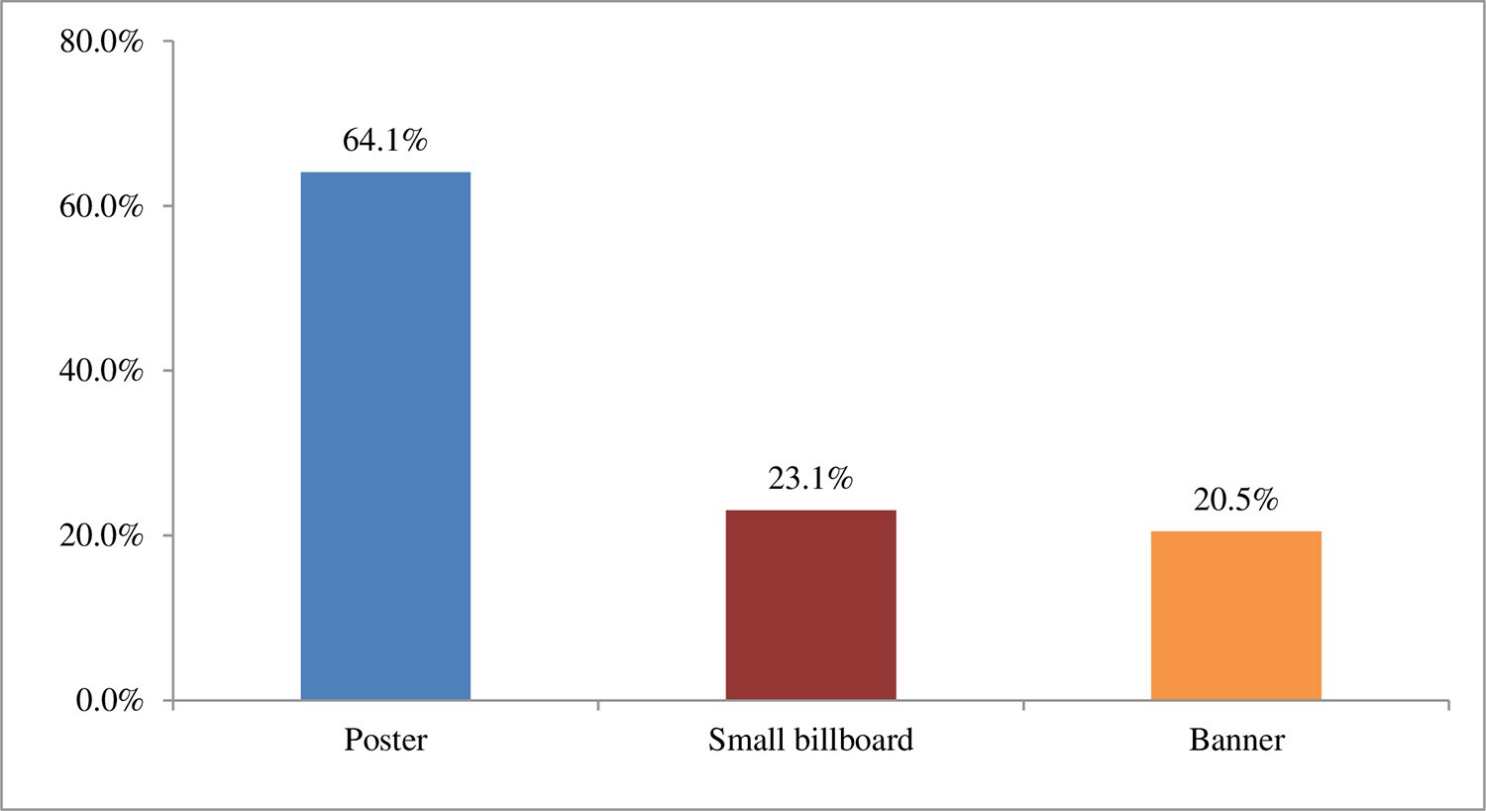
Types of advertisement of e-cigarette in the shop. The bar diagram illustrates the different types of advertisements displayed in the e-cigarette shop. It can be observed that the majority of advertisements were displayed as posters (64.1%). Additionally, 23.1% of the advertisements were displayed as billboards, while the remaining 20.5% were shown as banners.

**Fig 4:**
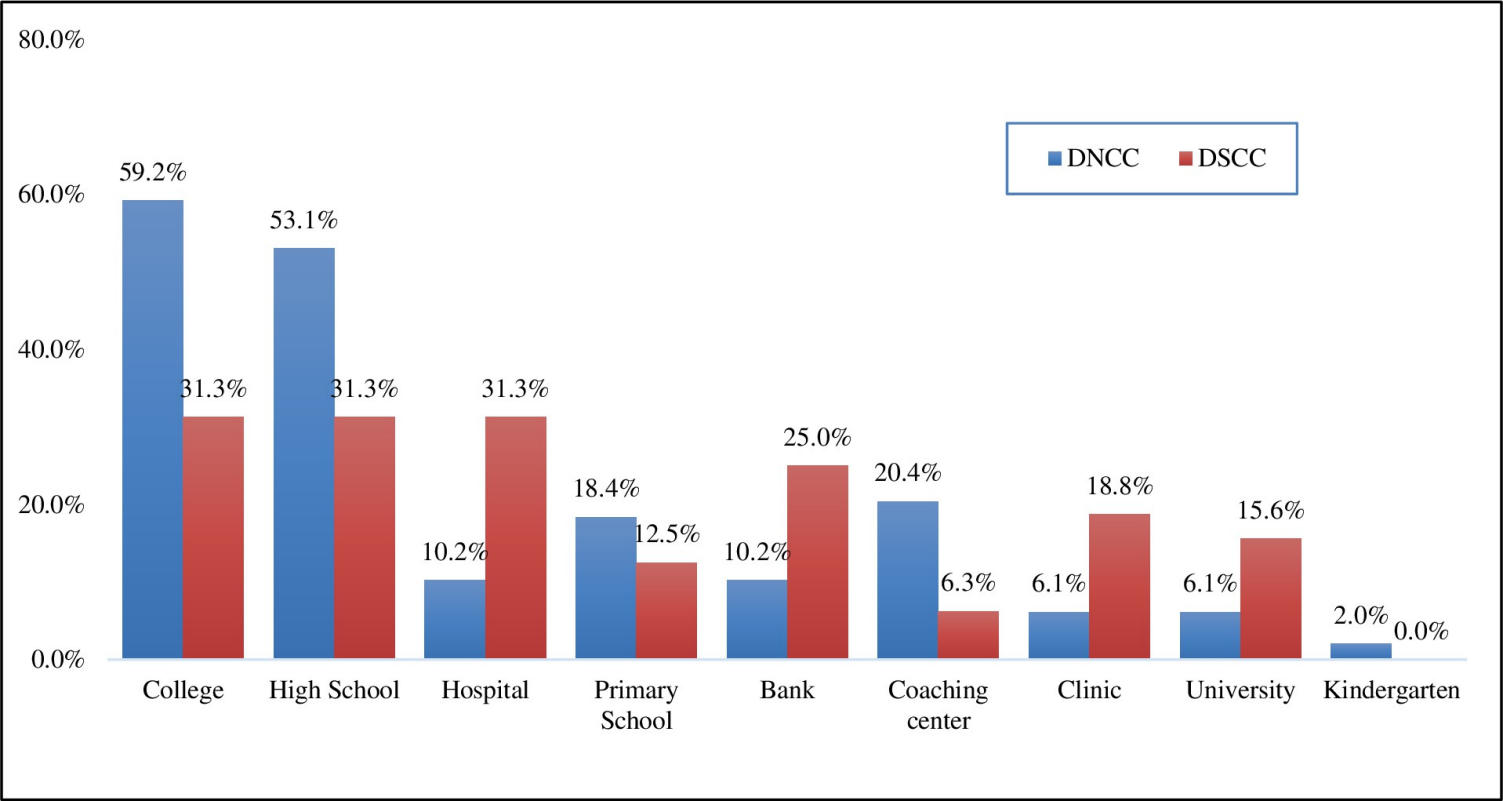
Institutions within 100 meters from the POS by city corporation. The diagram shows the percentage of different institutions within 100 meters of POSs according to observation. In Dhaka North City Corporation, the highest number of institutions that were found in front or within 100 meters of POS location were colleges (59.2%) and high schools (53.1%) respectively followed by coaching center (20.4%), primary schools (18.4%) etc. The lowest percentage of kindergartens (2%) was found near different POS locations. On the other hand, in Dhaka South City Corporation, maximum percentage of institutions was found within 100 meters of POSs were colleges (31.3%), high schools (31.3%) and hospitals (31.3%) followed by banks (25%), Clinics (18.8%) etc. while the lowest percentage was for coaching centers (6.3%).

The distribution of the location of POS based on City Corporation is depicted in Fig 2. It is observed that most of the POS located at shopping malls in both city corporations, whereas 55.0% in DSCC and 37.8% in DNCC followed by 5.1% (DNCC) and 2.5% (DSCC) in residential house, 3.8% (DNCC) and 0.8% (DSCC) in super shop (Fig 2). Moreover, advertisements related information has been depicted in Fig 3. In Table 2 presents the diverse types of institutions found within 100 meters of different POSs according to observation. Highest 39 (48.1%) colleges, 36 (44.4%) high schools and 15 (18.5%) hospitals were found within 100 meters of different POS locations. We got only one kindergarten within 100 meters of one of our POS locations. The diagram shows the percentage of different institutions within 100 meters of POSs according to observation. In Dhaka North City Corporation, the highest number of institutions that were found in front or within 100 meters of POS location were colleges (59.2%) and high schools (53.1%) respectively followed by coaching center (20.4%), primary schools (18.4%) etc. The lowest percentage of kindergartens (2%) was found near different POS locations. On the other hand, in Dhaka South City Corporation, maximum percentage of institutions was found within 100 meters of POSs were colleges (31.3%), high schools (31.3%) and hospitals (31.3%) followed by banks (25%), Clinics (18.8%) etc. while the lowest percentage was for coaching centers (6.3%) (Fig 4).

### 3.2 Geographical mapping

**Fig 5:**
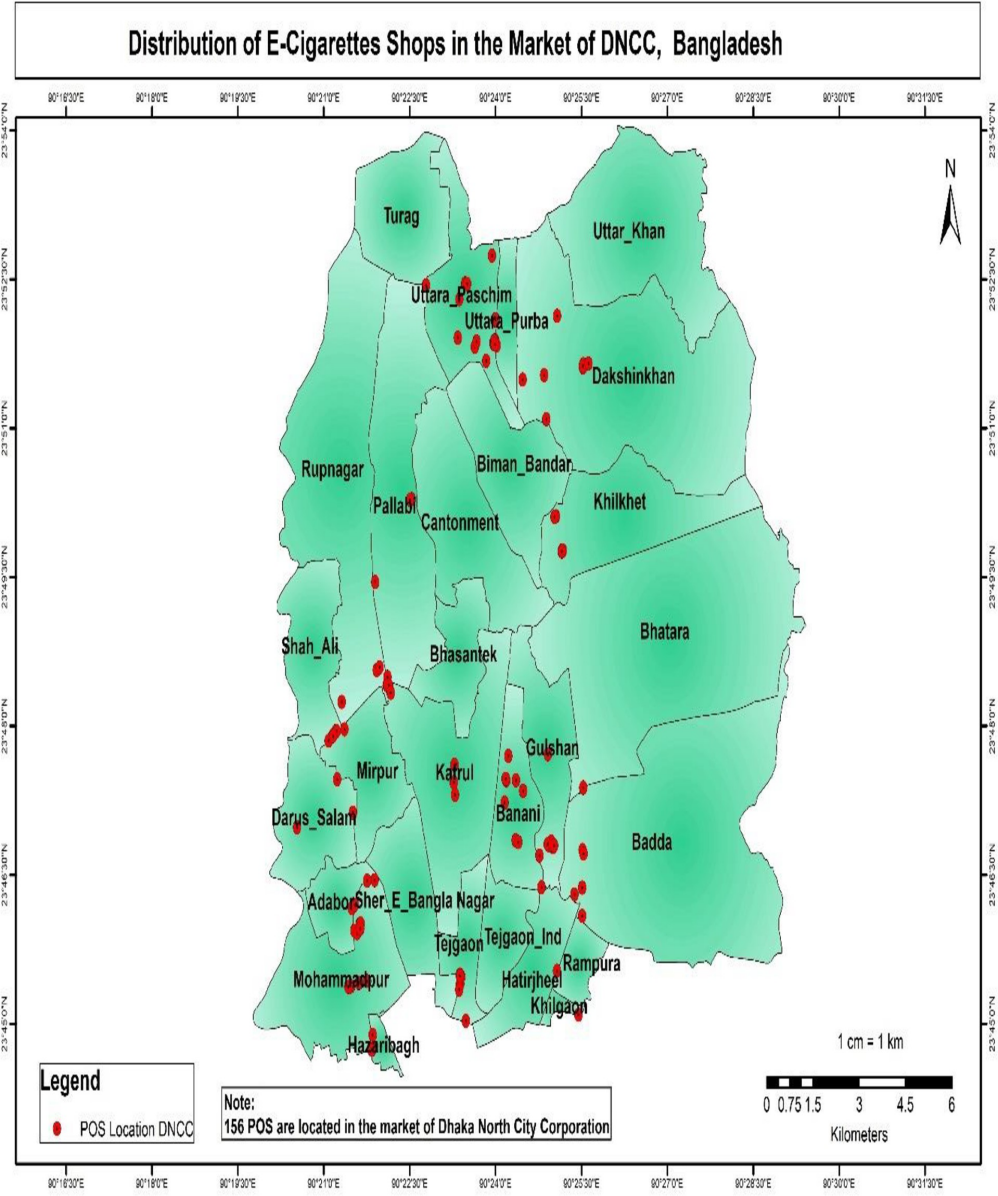
Distributions of e-cigarettes shops in the market of DNCC in Dhaka City. The results of the buffer and multi-buffer ring analysis are graphically represented on the map. The results provide a comprehensive analysis of the distribution of e-shops in Dhaka North City Corporation. From the map, it is evident that Uttara, Mirpur, Gulshan, Banani, and Adabor are the areas with the highest concentration of e-shops in DNCC.

**Fig 6:**
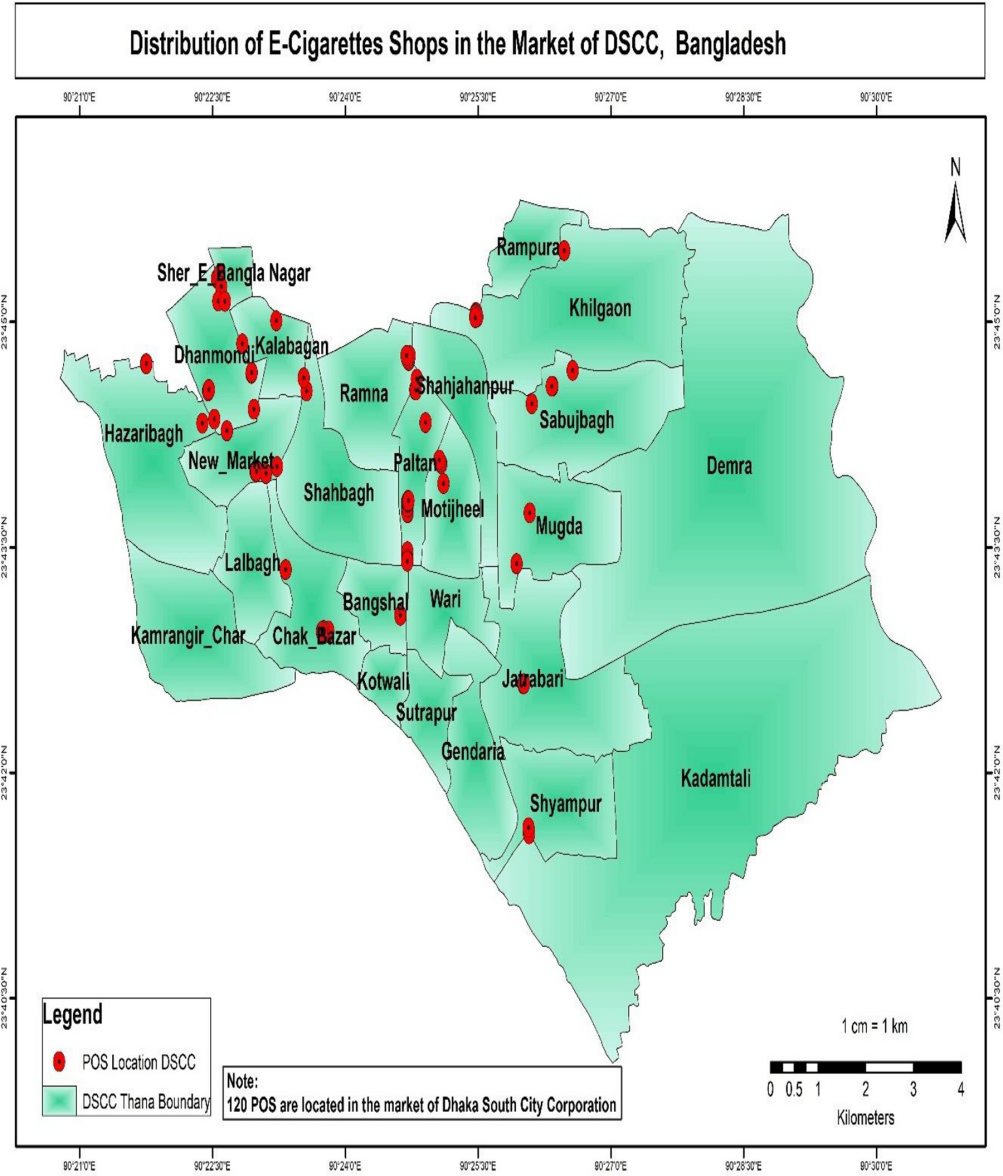
Distributions of e-cigarettes shops in the market of DSCC in Dhaka City. In Dhaka South City Corporation, New Market, Sher-e-Bangla Nagar, Dhanmondi, Kalabagan, Motijheel, Shahjahanpur, and Chak Bazar are areas with a high concentration of e-cigarette shops.

**Fig 7:**
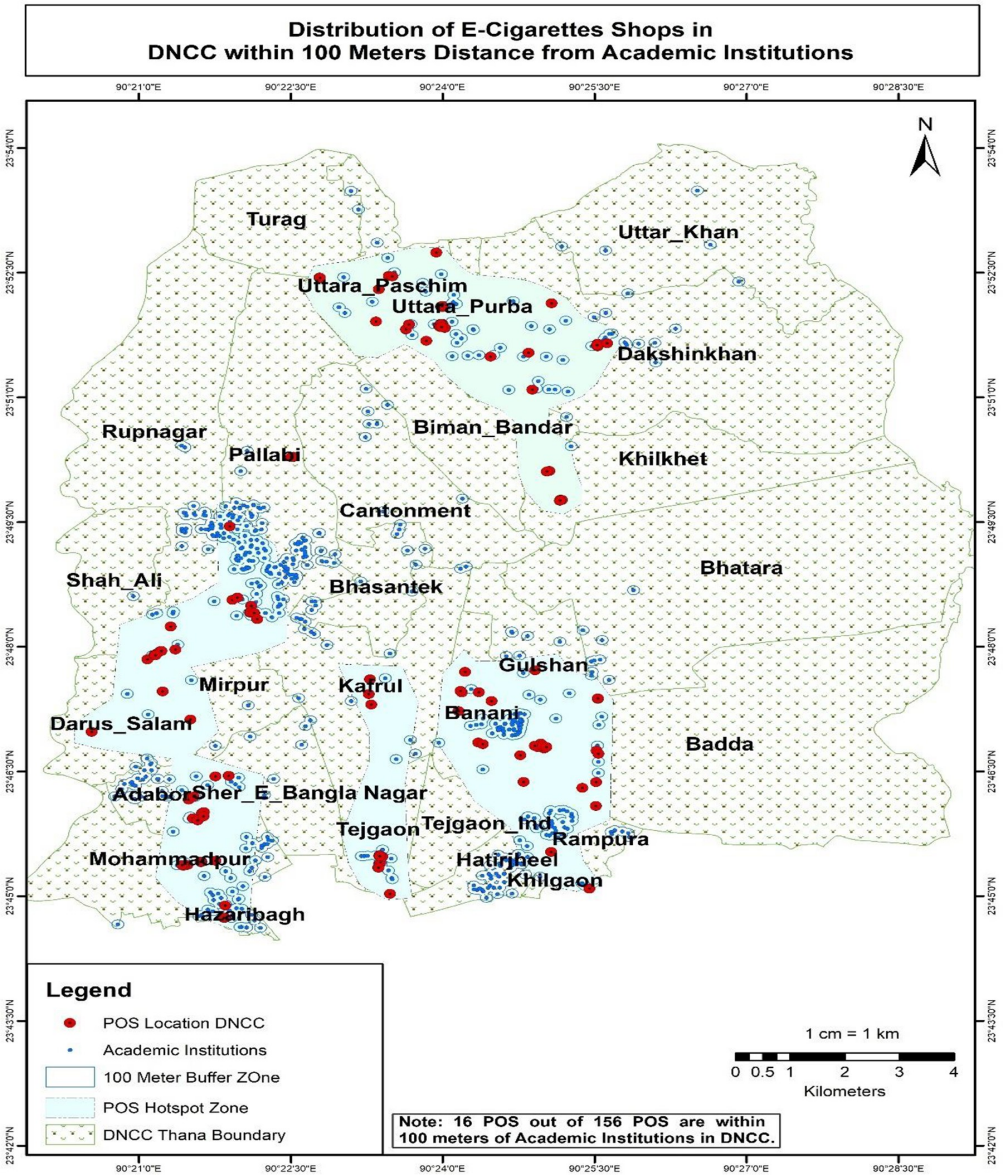
Distribution of e-cigarettes shops in DNCC within 100 meters distance from academic institutions. In Dhaka North City Corporation, it is identified that 16 e-cigarette shops are situated within 100 meters of academic institutions.

**Fig 8:**
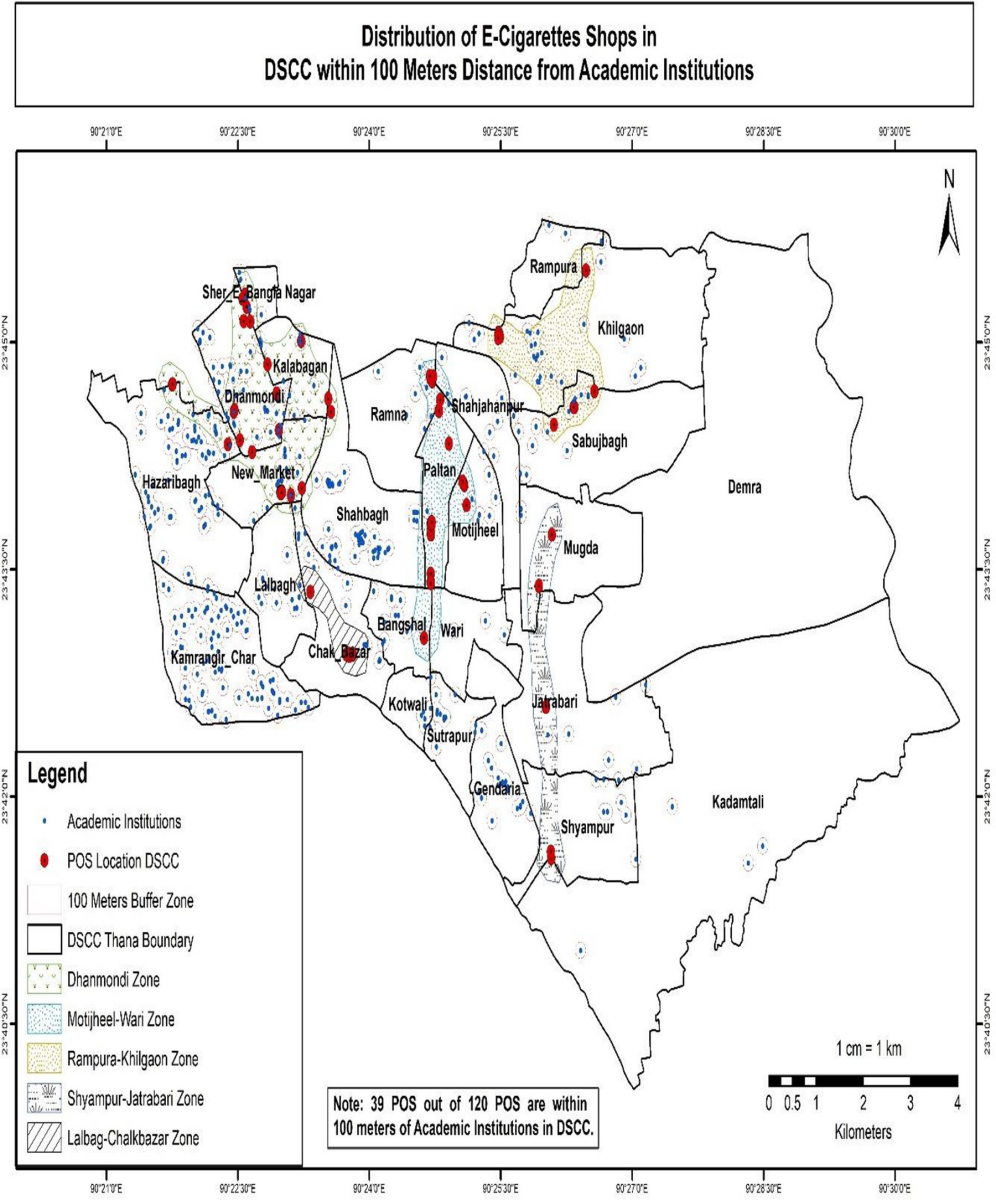
Distribution of e-cigarettes shops in DSCC within 100 meters distance from academic institutions. In Dhaka South City Corporation, there are 39 e-cigarette shops within 100 meters of academic institutions.

**Fig 9:**
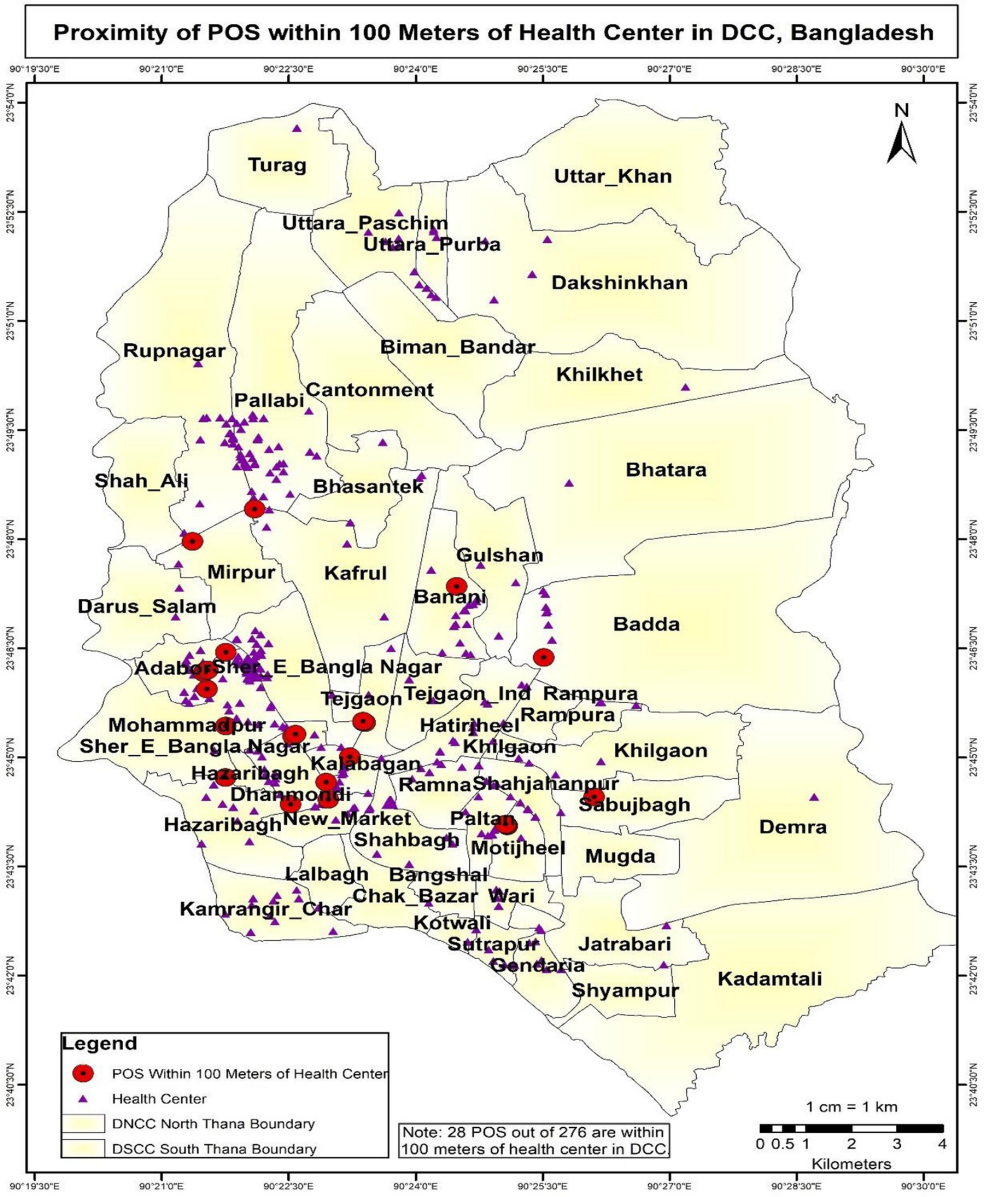
Proximity of POS within 100 meters of hospital in DCC. The e-cigarette shops located within the 100-meter buffer zone of hospitals are visually represented on the map. This visualization allows for a comprehensive understanding of the spatial relationship between e-cigarette shops and healthcare facilities. Finally, the maps generated from the spatial analysis are carefully interpreted and analyzed to identify the patterns of e-cigarette shop distribution and their proximity to hospitals. This analysis has significant implications for understanding potential health risks associated with e-cigarette usage in close proximity to healthcare facilities.

The results of the buffer and multi-buffer ring analysis are graphically represented on the map. The results provide a comprehensive analysis of the distribution of e-shops in both Dhaka North City Corporation and Dhaka South City Corporation (Figs 5 and 6) respectively. For instance, within Dhaka North City Corporation, it is identified that 16 e-cigarette shops are situated within 100 meters of academic institutions. Similarly, within Dhaka South City Corporation, there are 39 e-cigarette shops within 100 meters of academic institutions (Figs 7 and 8) respectively. The e-cigarette shops located within the 100-meter buffer zone of hospitals are visually represented on the map (Fig 9). This visualization allows for a comprehensive understanding of the spatial relationship between e-cigarette shops, healthcare facilities, and educational institutions. Finally, the maps generated from the spatial analysis are carefully interpreted and analyzed to identify the patterns of e-cigarette shop distribution and their proximity to hospitals and academic institutions. This analysis has significant implications for understanding potential health risks associated with e-cigarette usage in close proximity to healthcare facilities and educational settings.

### 3.3 Perception regarding e-cigarettes

#### 3.3.1 Perception regarding e-cigarettes features

The e-cigarette is a long pen-style rechargeable device, which requires a management system that is referred to as Power IC. E-cigarettes do not contain tar. It uses synthetic nicotine. Since it is vaporizing, it does not seem to be a big deal. It may have featured a coil system and was installable with a liquid that has a sticky appearance. Four components, Propylene glycol (PG), Vegetable glycerin (VG), flavoring, and nicotine must all be present in a juice to be used. Propylene glycol, sometimes known as PG, is a colorless, odorless liquid. Vegetable glycerin, on the other hand, referred to as VG has a little sweet flavor and a slightly thicker consistency. According to the responses of retailers:

*The electronic cigarettes were rechargeable and looked like long pens. It was compatible with liquid installations and included maybe coil system.” [R03.001]*

“*Regarding devices*, *the crucial elements are the battery*, *the Power IC for control*, *and the coils for activation*. *These coils are crucial for the process of converting the e-liquid into vapor*. *When it comes to e-liquids*, *there are four essential components that you need*: *propylene glycol (PG)*, *vegetable glycerin (VG)*, *flavoring*, *and nicotine*. *All four elements are essential for an enjoyable and satisfying vaping experience*.*” [R01*.*001]*

#### 3.3.2 Perception towards adverse health effects of e-cigarettes

E-cigarette retailers believe that these products pose fewer health risks than traditional cigarettes due to their adjustable nicotine levels and lower nicotine content. However, they also mentioned some negative aspects- such as water can lodge in the lungs causing blockages and leading to abdominal gas. However, if nicotine is consumed in excess, several issues may arise. For example, stomach issues, one also has headaches, sinusitis, and migraines. Additionally, it has a nicotine tobacco flavor, which increases the risk of heart attack, certain flavors are ice flavor, which causes a cold, and occasionally it affects the throat and causes cancer when used frequently. Regarding all of these issues, retailers have confessed that:

“*Everything has side effects. ome chemicals are safe to breath, while others are harmful to human health. Consuming fruit-flavored juice will not affect your health. Fruit-flavored juice will not harm your body if you take it. When you consume that clone juice you will be harmed. Because they do not maintain food grade. Burning anything produces a tar that is inhaled and causes bacterial infections, as well as cancer due to chemicals.” [R03.007]*

“*Blueberry drink is often unsuitable for a large number of people*, *who often choose not to consume it*. *However*, *few people complain of gastrointestinal discomfort from its use*. *Besides*, *I have not seen any trouble so far*. *If you smoke cigarettes for 90 to 100 years*, *then the risk of cancer is 100%*. *E-cigarettes are not carcinogenic*. *Cancer cells remain dormant in our body until they are triggered into activity by our unhealthy lifestyle choices*. *Subsequently*, *the cigarette is regarded as a medium that individuals believe is responsible for certain outcomes*.*” [R03*.*001]*

The key informants of our study talked about the health hazards associated with e-cigarettes. E-cigarettes contain non-natural substances, such as chemicals and juices, which can lead to various forms of cancer and affect the organs when burned. Other illnesses, such as asthma, may also manifest as well and chronic obstructive pulmonary disease may occur. In addition, e-cigarette use by children can hinder brain development and, with prolonged use, may lead to a decrease in intelligence quotient (IQ). Furthermore, long-term nicotine use is likely to raise our risk of heart attack or stroke due to the atherosclerotic disease process that nicotine causes.

“*E-Cigarette contains nicotine means e-cigarettes are harmful to health and I say e-cigarette is never a substitute for a conventional cigarette I say it is not safe. Formaldehyde and acetate are very harmful to the body. As I have said before, even in our developed countries, there is a lot of research on e-cigarettes. The Japanese say that this cigarette is 10 times more harmful than normal cigarettes, because of its use which may lead to the development of various diseases including stroke, heart attack, or cardiac attack. E-cigarettes are in no way less harmful than traditional cigarettes.” [KII-05]*

“*Long-term use of nicotine may lead to the development of atherosclerosis*, *a condition that increases the likelihood of heart attack or stroke*. *In addition*, *the presence of additional compounds in the substance might occasionally provide a danger of pneumonitis or lung infection*. *Furthermore*, *it is seen with many impurities due to which people have died*. *Nicotine is the cause of our addiction*. *It harms brain development when used by children*. *It may gradually decrease the level of intelligence if it is used extensively*.*” [KII-04]*

#### 3.3.3 Perception regarding at-risk population for e-cigarette usage

People between the ages of 20 and 35 are the most common users, and the younger generation tends to smoke it more frequently. The age range of female consumers is 18 to 40 years old, while that of male customers is 18 to 60 years old. Although children utilize it now, but the sellers do not support them in doing so. Since there are no rules there, children purchase from the sidewalk. Street vendors would profit more if they could sell more goods.

“*A mother of an intermediate student once came into the store complaining that her son was unable to concentrate on his studies without vaping. Another time, a 15-year-old boy arrived, and we advised him to start vaping after a few days. Later, the mother of the youngster arrived and said, "I’ll buy it for my son, so why should you have any problems?" [R03.007]*

#### 3.3.4 Perception regarding e-cigarette consumers

All types of customers come here but the sellers do not sell to kids. People their 25 years and 30 years old make up the largest attendance group. The younger generation makes the most use of it. Although people of other ages visit as well, teenagers appear to come mostly to purchase vape. Older individuals, including those in their 40s and beyond, also frequent the place. Both lone individuals and groups make up the bulk. Middle-aged individuals typically smoke a large quantity of cigarettes throughout the day. They then encourage their friends who smoke to transition to e-cigarettes rather than continuing to smoke. Thus, friends influence friends. More boys come, while the number of girls is very limited. School-age students now visit during tiffin breaks and smoke cigarettes in tea stalls.

“*The majority of my customers are younger buyers, particularly those under 15 years old. Most of them are students, with boys from well-off families above 20 years old also making up a significant portion of my customer base. Around 70% of my customers are boys, while the remaining 30% are girls. Many of the girls are regular customers, with 5–6 of them coming to buy regularly. They usually come alone rather than in a group, except for the 7/8-year-old school children who come in groups. Additionally, it is common for one friend to bring another friend to buy on different days.” [R12.012]*

“*The target audience is typically 18 to 24 years old*. *It’s becoming popular online*, *especially with active internet users who post cloud photos on Facebook*. *It’s a trending topic and can go viral” [RA12*.*001]*

“*Small children also come but we have fewer here*, *go to the shop in Kurmitola and many school and college students come to buy e-cigarettes*. *Kids get interested in e-cigarettes by seeing others using them or seeing e-cigarette devices*. *And the boys and girls of Dhaka are a little more updated*.*” [R04*.*016]*

#### 3.3.5 Reasons for liking e-cigarettes by consumers

Everyone knows that cigarettes are bad for people in this day, particularly in the age of social media. However, users of e-cigarettes prefer to consume them to stop smoking. They believe that e-cigarettes are a safer option. E-cigarettes are less dangerous when compared to traditional cigarettes as they do not produce Bad odors. Here, there is more flavor addiction than nicotine addiction. In addition, it has become a direct fashion, so everyone wants to try it. There is a growing trend among the younger generation to associate vaping with being trendy and cool. Unlike traditional cigarettes, which emit strong odors and are often not tolerated in many places, e-cigarettes can be used almost anywhere. But people can use vape everywhere. Due to having these kinds of advantages people like to use e-cigarettes.

“*The reason is flavor addiction. Nicotine addiction is less here, flavor addiction is more. When you have a mango flavor in your mouth all day long, when you have an apple flavor, it feels a little soothing. So, there are the main things, flavors. They come back for the flavor.” [RA08.001]*

“*Those who are over 30 years old are mostly smokers*. *They use vaping to abstain from smoking*. *People of this type mostly come to me*. *Some people come and tell me I want to quit smoking and suggest some flavorful vape*. *Some come here to buy vape due to the scent and flavor*. *I think most importantly people are using vaping as a substitute for smoking*. *If you light a cigarette near anyone*, *they think badly about them*. *If you do vape people do not think it is bad*. *It is seen that many have quit smoking due to vaping” [RA12*.*002]*

“*The first reason behind this is that the customers consider this thing to be safe by using it by them*. *They did not have any problems*. *In addition*, *as the way we told them to use it*, *they follow that*. *By doing so they enjoyed vaping” [R01*.*001]*

### 3.4 Distribution mechanism of e-cigarettes

#### 3.4.1 General distribution process of e-cigarettes

In Bangladesh, e-cigarettes are not manufactured by any company. Instead, e-cigarette importers bring in various vaping devices and juices from other countries. These products are then supplied to wholesalers, distributors, and retailers in Bangladesh. Retailers from dedicated vape shops, other physical stores, and online stores sell e-cigarette products directly to consumers.

“*I brought all kinds of devices from China. Although the original brands are from the USA and UK, the products are manufactured in China. In addition, some Swiss ones are made in Switzerland that are very expensive. But they do not come to Bangladesh now, they used to come earlier” (R03.001)*

“*All my juices here are from America and all devices are from China” (RO4*.*013)*

“*…These companies are foreign companies*. *In our country no vape is produced*, *vapes come from outside countries*. *We have no authority over this matter” (RA12*.*002)*

“*USA and China*. *I order the devices from China and liquid from the UK and USA*. *Liquid is also available in China*, *but we do not import” (R03*.*005)*

#### 3.4.2 The place of availability of e-cigarettes

Our study respondents have shared that Mirpur, Gulistan, Dhanmondi, Nikunja, Baridhara, Gulshan, Banani, Uttara, etc. are the major hotspot of e-cigarettes. One of our responses mentioned that there is a factory in Chakbazar, from which they get the e-cigarettes.

“*In terms of availability, I would say Dhanmondi. In addition, if you say in the case of use, then in that case I will say Gulshan or Banani” (R01.001)*

“*You will find it mostly in the Chalkbazar*. *If you want to see new types of vapes*, *you can visit Chalkbazar*. *They go wholesale there*. *Friendship market*, *10 no*. *Lane*. *You can find more information regarding vaping there*. *They buy thousands of pieces” (RA12*.*002)*

Respondents also stated that e-cigarette marketing is expanding in places where more wealthy people stay. According to them, many people are already accustomed to these types of cigarettes and these are becoming available in every shopping complex.

“*You can find them in New Supermarket, Mirpur, Elephant Road, and Bashundhara City. Vapes are now sold in every shopping complex as an essential item, just like rice and fish. Sometimes using vapes makes everyone look smart” (RA12.001)*

“*There are many shops in Gulistan*. *The residents of that area have more money*, *while the people in our area have less*. *It’s important to understand that there is a strong connection between the e-cigarette business and its location*.*” (R04*.*016)*

#### 3.4.3 Available replicas of e-cigarettes

Long-term vaping has been shown to have negative health impacts; its replicas may even be more detrimental. In the case of inquiry regarding the availability of replicas, retailers emphasized that they are selling the original product imported from abroad, but there are many sellers of e-cigarettes in Chawkbazar, Newmarket, and Old Dhaka engaged in selling replica products.

“*I do not know if it is in Bangladesh or not but maybe in China. Dhaka New Market and Street Shop sell clones/replicas; they seem to sell Chinese juice. I am selling American Juice” (RO4.013)*

“*I do not know that brother*. *It may happen*. *Nothing is impossible here*, *everything is possible*…*If you want to see them*, *then you will have to walk around the streets of Chawkbazar*, *Jinjira” (R04*.*016)*

“*Replica means disposable devices; there are some clones of disposables in Bangladesh*. *There are several clone products available in Newmarket*, *they import them from China” (R05*.*001)*

Retailers’ comments indicate that e-cigarette duplicates are produced nearby, notably at Chawkbazar. Although it is challenging to replicate the device, fake juice may be made cheaply and sold for a lesser price. Since many people cannot afford the original e-cigarette and its fluids, these could boost marketing among the general public. One person described the procedure for creating replica juices, which he had heard from people he knew.

*“However, apart from the authentic products, clone products are available in the Round Square under the foot over the bridge and in the new market with budget money. We sell 100 ml in 1800 to 2400 taka. They sell 30 ml for 300 taka, which we cannot imagine. Replica juice does not come from another country; they are made in our own Bangladesh. Made in Jinjira and sold in Chawkbazar. The whole thing is distributed from Chawkbazar. Pod devices are not usually cloned. They are difficult to clone. Mod devices are often cloned” (R03.007)*

#### 3.4.4 Marketing process of e-cigarettes distribution

Social media platforms like Facebook, Instagram, WhatsApp, TikTok, etc. are being used extensively as a way of buying and selling different kinds of products. Online marketing of e-cigarettes is also growing in Bangladesh. These also allow the sharing of necessary information regarding e-cigarettes, like their flavors, prices, promotional messages, delivery process, etc. Our study respondent stated that online media have made the e-cigarette business and delivery process easier as well. Online media, apps, and websites related to shopping, such as Daraz are also involved in e-cigarette marketing.

“*…Yes, Facebook, and Instagram. The page is mainly for online delivery of parcels. We don’t post videos on YouTube. Customers can place orders via WhatsApp and receive home delivery. We only deliver through Instagram if someone comments on the product’s picture or sends a personal message.” (R03.007)*

“*Through online*, *we do business through our Facebook page*. *We also have a website*. *However*, *if it is in the case of wholesale*, *then it is normal to talk on the phone*. *So roughly*, *you can catch these dealings through WhatsApp or over the phone” (R01*.*001)*

“*No*, *I do not give Ads*. *Daraz gives an ad… Yes*, *everything is available in Daraz*. *Over 90% of goods are sold in Daraz” (RA08*.*003)*

Moreover, some retailers also shared that in-store selling of e-cigarettes is more feasible for them. According to them, the promotion of this specific product in social media may become the reason for governmental sanction. Retailers have to keep themselves busy by selling different kinds of products at once in their stores. According to them, e-cigarette sales are more in their store than the online platform. Online business for e-cigarettes has become tougher for them, except for the dedicated e-cigarette shops.

“*Online promotion of tobacco products is prohibited by the government, and this also extends to social media.I do not know if it goes to foreign countries. Online sales for tobacco products are generally poor, as most transactions occur in physical stores” (R03.001)*

“*Long ago*, *I had a Facebook page called "Gazette King" where I posted pictures*. *Now I do not run it*. *My shop sells more than the page*. *Those who have free time can browse the page*. *I do not have that time” (R12*.*012)*

A few retailers acknowledged that they offer electronic cigarettes offline as well as online.

“*Both online order and direct selling… Since home delivery is an option, many people also want to visit the shop… I would say 20% is online. We have many in-shop customers” (RA08.001)*

### 3.5 Marketing strategy of e-cigarettes

#### 3.5.1 Physical and online marketing strategies for e-cigarette

To promote products retailers rarely use postering or other offline marketing. Dedicated shops for e-cigarette marketing use online marketing strategies more than that of physical stores that sell these devices as a side business. They depend on online marketing strategies more and advise customers to see or give reviews on Facebook and other business apps. Discounts are offered occasionally, but it is not maintained according to any specific product or occasion. First-time customers are given some benefits. To generate interest in their products, they have a range of eye-catching e-cigarettes featured on their website (also, their Facebook page features numerous images of e-cigarettes) for their eyes to perceive the attraction. In addition, they just say they will sell it at a low price, even if the customers take the flavor they will sell it also at a low price. The people who see the product know the value, and that is why they buy it. Therefore, there is nothing to make lucrative here. They try to give advertisements, but there is a risk of banning the page. Customer satisfaction and taste addiction are also factors. There has been observed an alluring representation of different types of devices in the store. Retailers also use verbal explanations and motivation, which convince them to buy e-cigarettes. Some statements of respondents are given below:

“*I always ask customers about their vaping needs, whether it’s for creating big clouds or to help quit smoking. Based on their response, I introduce them to different products. I had mentioned earlier that e-cigarettes can be harmful, although less than traditional cigarettes. We tell people that it is also harmful to those who do not know. Keep a few interesting e-cigarettes now and then. We do not show. We put a variety of interesting e-cigarettes on the online page (Facebook page) just to make people interested that we have some interesting vapes in our store. So that the attraction works in their eyes” [R03.001]*

“*I will sell it also at a low price*. *There was a separate display case for vaping on one side of the shop*. *A customer was shown an interesting vape device that looked like a pistol” [R12*.*012]*

“*It is more or less*, *everyone involved in marketing*. *Anyone who has any stock is taking pictures and just sharing them*. *Just this*. *Because of the way our vaping thing is*, *we do not need to make it lucrative here*. *Many people recognize the value of our product and that’s why they buy it” [R01*.*001]*

During the pandemic, companies have provided e-cigarettes to households via social media. Therefore, since there is no law, they take advantage of every tactic at their disposal to attract customers. They have chosen to target a specific age group, especially the young generation, so that is where they will draw it out and brand it to sustain the business for a long period. After conducting research, the tobacco businesses discovered that lowering the price is necessary to reach more individuals. They are using a technique to lower the price because of this. They might be strategizing ways to reduce the cost of the products. The market will grow as more people become dependent on it. In addition, in the drama industry, the producers and directors receive financial support to maintain scenes featuring smoking electronic cigarettes for promotional purposes.

“*The marketing strategy seems to involve targeted social media campaigns. Users who are in the young group are sent targeted messages with information and in terms of marketing they use online marketing mostly and now it is available in different stores and different markets” [KII-04]*

“*I have seen that they have targeted an age group*, *meaning the target group is where*, *even under the grave*, *they will pull it out and then brand it*. *Therefore*, *not only an educational institution but also the age group*, *that age group is their target*. *They grab a group between the ages of twelve and twenty*. *So*, *if they can make them addicted*, *I said they will do business for up to 75 years*. *They will not show much interest in an old 55–60-year-old person like me*. *Since I could pass away before I turn eight or ten years old*. *There is no profit in investing in this business*. *So*, *they target the place where that age group is available [KII-01]*

“*The tobacco companies have researched and found that if it is to reach people*, *they have to bring down the price*. *That is why they are bringing down the price by strategy*. *They may be planning how to make the devices cheaper*. *They have a plan*. *Nothing happens to them without planning*. *When he becomes dependent*, *the market will expand*. *It may be*, *now their sales are maybe 10*,*000 per year*. *So*, *if the price is reduced it may increase the sales to 20000 per year” [KII-02]*

“*It seems to me that the promotion is an increase in dramas*, *which was not before*. *Coca-Cola spent a lot of money to feature the brand in an Aamir Khan movie*. *So now*, *if they show smoking*, *maybe they take at least a few cores to show a smoking scene for only 10 seconds to 20 seconds*. *These are industry promotions*. *In drama*, *it is the producers and directors who are given money to keep this scene in our drama industry promotion*. *And especially if there is a popular actor-actress*, *and in our OTT platform*, *I do not see that much*, *but I have heard from people who study that the hero is in a bad mood*, *takes e-cigarettes*, *drinks*, *takes cigarettes*, *even when he is in a good mood*. *The matter is like that” [KII-03]*

#### 3.5.2 Motivational approach for the buyer to sell e-cigarettes

The sellers emphasized promoting e-cigarettes by expressing that, these e-cigarettes can cause some harm, but they are still less than traditional cigarettes, which is yet unproven. The customers unaware of those statements become well-informed, while the aware do not need an explanation beforehand to buy e-cigarettes. They offer options to customers and give them a polite explanation when they arrive. When a consumer visits, the sellers first try to understand their preferences and reasons for vaping. Customers who were trying to give up smoking were assisted in selecting juice.

“*Normal cigarettes have bad smells. Maybe that causes them problems. Their wives may scold them for smoking. If they use vape, they will not have any problems. At home, they can use vape too.” [RA12.002]*

“*When a customer comes to us*, *we first try to understand his preferences and why he wants to vape*. *If he comes to quit smoking*, *we help him choose juice*.*” [R05*.*001]*

“*In my opinion*, *if you like it*, *then go ahead and take it*. *When you take it*, *the smoke will come out*, *but if you don’t take it*, *the smoke won’t come out*. *This is just the approach*. *I do not tell someone to buy it*. *I do not tell them it is good*, *take it*.*” [RA08*.*003]*

#### 3.5.3 Promotional statement to increase sales of e-cigarettes

E-cigarette sales are largely dependent on liquid and device quality. Only those who are aware of e-cigarettes purchase the devices; others do not. On Facebook, the sellers do only post. The vast majority of the sellers do not write sales-oriented statements outside the shop. Their clients handle the majority of their marketing. Because a satisfied customer will refer five more customers to purchase the product. Therefore, the sellers do not need that type of marketing that much.

“*It has to be said that the customer will not understand which is good and which is bad. Flavor, device, price, and quality are different. It is better than smoked cigarettes, I usually say. If the smoke is more, the quality will be better. If the coil is damaged, you can change it here. You will get liquid of many flavors, I will keep less money, its quality will be better—this is what I say” [R12.012]*

*“I will not say any such slogans or statements*. *Slogan base statement we will give when we protest it*. *We do not need to protest*. *We are doing something good*. *If you want to join us*, *you can join*. *There are many anti-alcohol protests*, *but there are a few people here who are anti-alcohol*. *So*, *we do not have slogans for these things*. *We are providing safer options if you can join you can join*. *See how you like it*. *If you like the culture*, *then you can live with it” [R01*.*001]*

#### 3.5.4 Several ads related to e-cigarette marketing

Retailers stated that they do not offer any advertisements about the marketing of e-cigarettes, although they have been noticed on Facebook sites, YouTube, and Instagram. Facebook features advertisements, and users have the option to see reviews and watch videos on vaping. Sometimes, Daraz occasionally runs advertisements.

“*No, brother, currently YouTube is your platform for giving reviews. We mainly operate through reviews. For example, we recently reviewed Jellybox, and we set the Google price at 2600, but we sold it for 2400. We offer discounts on each product” [RA12.001]*

“*Every shop has signage outside*. *Like we have the ‘Vape in’ (shop name) logo outside in large form*. *So*, *we keep all branding up to the shop*. *Yes*, *there is a review system*. *However*, *this culture has not yet developed in Bangladesh” [R01*.*001]*

Among them, one of the people shared his experience in another way as if he had seen an ad regarding this:

“*I have seen an ad for another shop. Like a few days ago, “BARBIE” came out. Like this they took a picture which has “TANBIE” with the picture of the vape” [RA08.001]*

#### 3.5.5 Written statement in advertisements

The majority of the sellers do not give any written advertisement. People do not post product details that much now unless it is a second-hand selling post. Maximum people know about vape, in that case, sellers give the name of the product and the flavor of the juice. If customers want to know the rest, they can search on Google.

“*No (Smiling). I have never seen an advertisement for e-cigarettes.” [RO4.013]*

Some of them shared that some written statements they use for marketing like- It is less harmful than smoky cigarettes, waking up and smelling vape, and so on.

“*Coil it, wick it, juice it, vape it; Eat, sleep, vape; It’s glamorous on film but it’s not glamorous, waking up and smelling vape like an ashtray; Keep calm and vape like an ashtray; More stronger, more tasty; Under age 18 is prohibited” [R03.007]*

#### 3.5.6 Giveaways for retailers to promote e-cigarettes

Regarding giveaways for retailers to promote e-cigarettes, the majority of the sellers shared that they do not get anything regarding this, if a device breaks, it is the vendors’ fault, and they are not even eligible for a refund. However, gifts are provided when something is sold in the market as well and those who import it may get it.

“*The thing is that vape is now sold at large. No need for them to give discounts. The wholesalers buy them from foreign countries. If they buy them at a cheap price, they can give some discount. Getting a discount from the company is not possible. These are for limited profit” [RA12.002]*

“*I used to get it*, *but now I do not get it*. *When a new design or model of a product comes*, *they would give a new model along with the previous product model*. *They said that a new one has come and please check the device without cost*.*” [R03*.*001]*

#### 3.5.7 Steps taken by retailers to increase e-cigarette sales

Regarding sales, there is a single computation. Sellers might increase sales by lowering their prices. Because not everyone can afford to purchase vaping items at all. The internet pages must be visited, posts must be created, and they must be promoted to enhance sales. Opening more locations in well-located retail centers will be necessary to boost e-cigarette sales. The difference is in how things work offline and online. Selling products online has the potential to boost sales. People prefer to purchase more things when they watch the advertisement. Additionally, there are cash-back incentives, which is a wonderful addition.

“*In order to boost sales, we need to open online pages, create posts, and promote them. While there are some complications, they are manageable, and the main challenge is the time commitment. The sale of any item depends on marketing, if the marketing is good, the sales will increase.” [R04.016]*

#### 3.5.8 Future plans of retailers to increase the e-cigarette marketing

Despite the high costs, they wish to open a new branch. If the system for cash back is implemented, it will benefit them. But if it is all governed by the established guidelines and oversight, they will also help to promote it. The uncertainty surrounding the government’s recent declaration regarding e-cigarettes is a cause for alarm, though.

*“First we have to create the market, we have to expand the business, and then we have to do the promotion. Now it is too late to bring the product. However, when everything is regulated by an official organization, everything will be under supervision, and then there will be demand. I will get the product quickly. Then the market will be created.” [R03.007]*

### 3.6 Planning on governments’ future banning approach to e-cigarettes

Regarding the views of the sellers, rather than prohibiting it, the government should promote it. The sellers have no control over it. If the government bans it, they will do other business. Alcohol and cigarettes are legal. Even if the government forbids them, they are powerless to challenge its decisions because their decisions are final. After that, they will stop selling it. Apart from banning e-cigarettes, smoking should be outlawed at the same time. Again, British Tobacco has been pressuring the government for several years to ban e-cigarettes. Because e-cigarettes have caused a decline in their sales in the market. However, the seller plans to tell the government, that if e-cigarettes are less harmful than normal cigarettes, then they should not stop them.

“*if the government closes it, I will do another business. If it closes, there is no problem; there is no lack of business. And I am a small one and those who have big businesspeople will think about them.” [RO4.013]*

“*If they ban it*, *we will be at a loss*. *It will be better if they do not ban it*. *People of all ages can use it*. *And cigarettes cannot be used everywhere*. *E-cigarettes can be used everywhere*, *and smokers of all ages can use them*. *There is no obstacle*. *If you stop it*, *the bad people*, *the drunk party people*, *will get more addicted to other substances*. *If they vape*, *the risk will not be so much*.*” [R12*.*012]*

One of them also shared regarding governments’ approach to forever banning e-cigarettes in the future in another way like

“*They (government) have tried. But we have powerful people. We will fight to continue it. Because I know cigarettes are bad. Now if the government of Bangladesh wants people to die by smoking cigarettes that is fine. We will try our best.” [RA08.001]*

### 3.7 Challenges in taking steps towards e-cigarette banning

In response to inquiries, key informants of our study have shared some challenging issues for E-cigarette banning in Bangladesh. One of the main obstacles to limiting the marketing of e-cigarettes is their addictive nature, which stems from their thousands of flavors and appealing designs. Some of the respondents claim that those connected to the e-cigarette wholesale industry could obstruct the enactment and maintenance of e-cigarette control legislation. Furthermore, a few massive tobacco industries may have influenced legislators. Nevertheless, to expedite the e-cigarette ban, the health ministries must work harmoniously with other ministries in an integrative manner.

“*The business of addictive products is enormous. As a result, consumers who purchase these products often behave irrationally. Their behavior is not rational when it comes to buying these products. Even when they know that addiction is a risk, the price does not deter them. Their addiction is driven by the product’s flavor. Despite being aware of the negative consequences, they continue to support the business. These consumers are so heavily influenced that they cannot grasp the logic behind their actions. It all boils down to the money going into the pockets of those behind the business, which the consumers willingly spend.” [KII 1]*

“*Regarding the challenge of the ban*, *I said that those who are very normal*, *those who are associated with this business*, *those who are planning this business*, *and those who will produce it in this country*, *want this law not to be passed*, *maybe they will delay this law*. *They will do two things*, *first not to pass*. *Compromise will come in the second stage*, *weakening it a bit*. *What do you mean weak*? *They will say that it is not a ban*, *they will talk about control*. *As we call the Tobacco Control Law*, *so is the E-Cigarette Control Law*. *That is how to control it”*. *[KII-1]*

“*The first is that tobacco companies are giant companies*. *They influence everything from the policy level*. *Therefore*, *this is a big challenge*. *And their tactics are bad*, *they create influence where there are policymakers to motivate them in different ways*. *So that is a challenge for us*. *Another big challenge that we face is that we policymakers need to look at these issues from a public health perspective; as this is a public health issue” [KII2]*

“*It seems that there are significant challenges related to industry restrictions*. *The Ministry of Health has tried to initiate changes to the law in order to protect public health*. *However*, *there is a lack of support from other ministries such as the Ministry of Industries*, *Ministry of Agriculture*, *Ministry of Finance*, *and NBR*. *It appears that these ministries prioritize financial gain over public health*, *making it difficult to enact the necessary changes*. *This lack of support has ultimately prevented the correction from taking place*. *It seems that there are internal barriers preventing the progress of these crucial amendments*. *[KII 3]*

### 3.8 Available programs, rules, or policies against e-cigarette

For Bangladesh’s e-cigarette ban to be successful, it is crucial to implement specific measures to oppose the extensive marketing and promotion of these items. Currently, there is no law in Bangladesh regarding e-cigarettes. Based on the responses from our key informants, we know that campaigns against e-cigarette banning have been going on for more than three years. Furthermore, the government of Bangladesh has already drafted a bill that will shortly be approved by the parliament. Additionally, there is a variety of non-governmental organizations and anti-tobacco organizations working on awareness campaigns about these problems.

“*There is no law, there is nothing. We tried to say, it should be banned. And the campaign is going on for 2–3 years… and the government has decided in principle, a draft has been prepared. Now it was supposed to be raised before this election in the cabinet, no, after the election we hope, if there is no major interference, it will be passed here and then it will be passed in the parliament. Therefore, this has been a movement from civil society for a long time. The Government is also motivated, the Ministry of Health of the Government has made a regulation” [KII 1]*

“*There are different foundations they create front groups to bring these things*, *create business groups like we all know “Smoke-Free Foundation” they promote this e-cigarette all over the world as harm reduction” [KII- 4]*

“*Not only NGOs*, *all the anti-tobacco organizations in Bangladesh are working on it*, *that it is harmful to the body and health*, *that it should be banned*, *all the anti-tobacco organizations in Bangladesh*, *not only a particular NGO*, *BCCP*, *who give you these grants*, *also work on it”*. *[KII 1]*

“*We held press conferences*, *rallies*, *discussion meetings*, *and wrote different articles*…*Our organization has a Facebook page called Tobacco Free Bangladesh*. *If you go there*, *you will see that we posted about this cigarette*. *We also have a YouTube channel called DAM-Health*. *There we made a video about e-cigarettes and gave a message to make people aware*. *We re-share those videos on Facebook*. *In this way*, *we are making people aware… At the national level*, *not only us but many organizations*, *and some other anti-tobacco organizations have conducted programs on e-cigarettes*. *However*, *at the local level*, *I do not think anyone is doing it at the district level or upazila level*. *Because till now it remains a national issue because there is no law*.*” [KII -2]*

“*No*, *there is no finalized law and policy in Bangladesh so far*. *The Tobacco Control Act of 2005 has been amended once in 2013 having some loopholes*. *The revision work is still ongoing*. *This has been proposed in this amendment of the Act… Organizations working in the tobacco control community*. *As I work for people many other foundations work for people*. *Like the National Heart Foundation and Ahsania Mission*, *we have some alliances with doctors and the media*. *E*.*g*., *Bangladesh Anti-Tobacco Alliance*, *and Anti-Tobacco Media Alliance*. *Some such alliances and some individual organizations and individuals are doing the work regularly” [KII-5]*

### 3.9 Suggested initiates to ban e-cigarette

We can incorporate certain important steps to regulate or restrict e-cigarettes in Bangladesh based on the key informant’s responses. Its importation must be halted under the particular HS code. Strict regulations should be in place surrounding the marketing of e-cigarettes, and those who sell these products without a license must be compensated. The purchase of electronic cigarette devices should be restricted based on age and information about the harmful health effects of e-cigarettes. Since children and adolescents grow up in monitored environments, we can make sure that their use of e-cigarettes is strictly prohibited. The sale of e-cigarettes in the stores next to various educational institutions ought to be prohibited. Since e-cigarettes are portrayed as a healthy substitute for regular cigarettes, we can raise consciousness about the negative health impacts of e-cigarettes and encourage smokers to pursue other nicotine replacement therapies.

“*The first thing is that importation with HS code should be stopped. Imports should be stopped and then other routes that come in should slowly tried to be stopped. Apart from that, to stop the distribution, if you give the facility to import with HS code, then to distribute it—it is a legitimate product, anyone can distribute & sell it as they can. We did not arrange the license or anything like that.” [KII 4]*

“*Already e-cigarette has been banned in India… It is prescribed as a prescription drug in the UK*. *Those who wish to quit at once and it is not being given to anyone under 18 years of age… meaning there is an age limit*. *In America*, *they have made a rule that no flavor can be given*. *It is only given to those who want to quit tobacco*, *but they have banded off the flavors that would have been given to attract children*. *Besides*, *many other countries have completely banned it*. *At the local level*, *it should strictly ensure that children do not come to school with this item…Now the average initiation age for e-cigarette sage in Bangladesh is 15–16 years old… Doctors also are prescribing because it is available in the market*. *If it becomes illegal then there is no chance to prescribe…We have nicotine replacement therapy for nicotine*, *nicotine gum*, *and nicotine lozenges…” [KII 4]*

“*I have no idea about reducing distribution*. *I have no idea about the distribution mechanism*. *It has to be Law/Policy Implementation*. *The sale of e-cigarettes under the age of 18 cannot be implemented in our country*. *We have to ban it…import*, *export*, *production*, *marketing everything is prohibited…All types of production*, *marketing*, *sale*, *purchase*, *and marketing should be banned*. *Besides*, *we cannot give a healthy Bangladesh to the next generation*. *Our present people who are in policy-making*, *will not be alive anymore*, *they are supporting such activities and misdeeds for their benefit*. *Those who come after that will be victims of it*. *And many people will get sick*, *the young generation will suffer*, *and it will drive them to other addictions*.*” [KII-3]*

## 4. Discussion

This study provides insights regarding the availability, distribution, marketing strategy, and possible ways to reduce e-cigarettes in Bangladesh. First of all, the participants of the study perceived e-cigarettes as long, pen-shaped rechargeable devices that may have included a coil system and installable liquid with a sticky appearance that uses synthetic nicotine and does not include tar, which supports earlier research showing that e-cigarettes are battery-operated devices that heat and aerosolize "e-liquid." [39], and which requires inhaling vaporized nicotine during consumption [40], and the dangerous ingredients found in regular cigarettes, such as tar and carcinogens, were absent in e-cigarettes [40]. This study also shows that there are two main differences between traditional cigarettes and e-cigarettes: conventional cigarettes contain more nicotine than e-cigarettes, but e-cigarettes do not contain tar, which can lead to cancer. According to a previous study, it was found that many people believed e-cigarettes to be healthier than traditional cigarettes because they do not contain tar, smoke, or carcinogens [40]. Some participants also noted that e-cigarettes have lesser side effects than conventional cigarettes and help to normal cigarettes cessation. These findings are consistent with a previous study [41]. Regarding e-cigarette health impacts, our research found that besides serious issues like blockages, water can lodge in the lungs and produce abdominal gas. Additionally, using e-cigarettes may result in headaches, migraines, sinusitis, and gastrointestinal problems. It also contains nicotine tobacco flavor, which raises the risk of a heart attack; some varieties, like ice flavor, give you a cold. Sometimes, if you use it repeatedly, it causes cancer in the throat. The majority of the results are consistent with other researches showing that e-smoking can increase the risk of lung cancer and induce symptoms like nausea, vomiting, headaches, dizziness, choking, burn injuries, irritation of the upper respiratory tract, dry cough, and dry eyes and mucous membranes [42, 43]. Some of the participants in our study stated that e-cigarettes are more frequently smoked by younger individuals and their age range is between 20 to 35. Girls are extremely rare, while boys are more prevalent. Prior studies also showed consistent results, younger age groups are more likely to have ever used or are now using e-cigarettes [44]. Use of e-cigarettes is higher among boys but girls are increasingly at risk [45]. Some participants of this study also reported that children buy from the sidewalk because there are no restrictions there. However, there are few studies to compare these findings with therefore more extensive research is needed to bolster these conclusions. To guide future research on e-cigarette, use among teenagers and young adults and to inform policymakers and consumers about making evidence-based decisions, a thorough grasp of this literature is necessary [46]. Young individuals who use e-cigarettes run the danger of using cigarettes in the future. Since banning tobacco products has been shown to lower youth smoking rates, strict regulations pertaining to e-cigarettes are necessary for prevention [47]. Our findings revealed that e-cigarette importers usually import different types of vaping devices and juices from abroad. Similar findings were found, most of their e-cigarettes device and liquid come from abroad in Norway [48]. Our study respondents shared that Mirpur, Gulistan, Dhanmondi, Nikunja, Baridhara, Gulshan, Banani, Uttara, etc. are the major hotspot of e-cigarettes and some retailers’ sale replica of e-cigarettes in some place. To compare our findings there has not been found any previous study regarding this result. Our study revealed that e-cigarette distribution mostly occurs on online platforms such as Facebook and You Tube, this finding aligns with previous studies conducted in the United States. The majority of consumers had bought tobacco or e-cigarettes through online platforms, and both online and offline buyers noted that price and convenience had influenced their decision to buy tobacco or e-cigarettes from the online sites [49]. Furthermore, social media platforms serve as the main channel for e-cigarette distribution in Thailand [50]. Some participants in this study also highlighted that sometimes discounts are available, and many of the individuals who see the goods are aware of their value, which is why they purchase them. Moreover, addictive flavor and customer satisfaction are always in demand among customers, and if they can offer an outstanding flavor, it will work better to promote the e-cigarette brands. These findings are also consistent with a study [49] which found that there are four main reasons why people buy e-cigarettes online: price (cheaper online, discounts, buying in bulk); product characteristics (quality, availability); buying experience (time, convenience, reading reviews); and curiosity. In this study, regarding motivating the buyer’s approach to selling e-cigarettes, the retailers provide options to clients and greet them with courtesy upon arrival with a polite explanation, and customers attempting to quit smoking receive assistance in choosing juice. It also aligns with another study that found that individuals preferred to make purchases in person due to the opportunity to visually see, physically experience, and handle the goods, as well as receive guidance from store in person [49]. However, the quality of the liquid and device has a major impact on e-cigarette sales. The devices are only available to people who are aware of e-cigarettes; others who are not or do not buy them. The customers they serve do most of their marketing. The retail channel is the primary means of marketing cigarettes, as evidenced by a prior study that showed the bulk of cigarette companies’ advertising and promotional budgets were allocated for its distribution [51]. However, further extensive research is needed to address the lack of available literature on promotional statements regarding e-cigarette sales. Moreover, the majority of sellers, according to the report, do not provide written advertisements. When merchants disclose the product name and juice flavor, the majority of individuals who are aware of vaping use Google to find out more information regarding this and they also find reviews and videos on vaping on Facebook or YouTube. At the same time, vendors give some promotional speeches pleasantly related to the marketing of e-cigarettes. An earlier study showed that e-cigarette businesses’ marketing tactics have been linked to the rise in young users and video advertisements on digital platforms were found to be the most prevalent approach [52]. A previous study [53] also revealed that, using terms associated with the promotion of e-cigarettes, YouTube has been utilizing for this and consistent with this study. This study also found that retailers may boost e-cigarette sales by reducing their prices. When customers see advertisements, people tend to buy more products. This finding is in line with an earlier study that also found that sponsored events and social media advertising might be crucial parts of an effective e-cigarette marketing plan to boost e-cigarette sales [54]. Additionally, the offering of cash back incentives might serve as an effective strategy to enhance the sales of e-cigarettes. This study also made clear that every individual has a unique profit and sales volume, which vary based on where their stores are located In our study the majority of interviewees disclosed that they did not receive anything from the wholesalers or merchandizers; even if a device malfunctions, it is the vendors’ responsibility, and they are not even qualified for reimbursement. Since this was not included in the earlier research, a large-scale mixed-method study ought to be carried out to make this finding more generalizable. Nonetheless, the majority of vendors are happy with their businesses, and they want to launch new branches despite the high startup costs. They will also aid in its promotion if everything is in their favors. The uncertainty surrounding the government’s recent declaration regarding e-cigarettes is a cause for alarm, though. A previous survey also showed that a large number of states and towns have changed their smoke-free legislation to restrict public usage and ban the sale of e-cigarettes [55]. For instance, it appears that while they are happy with the sales of e-cigarettes, the prospect of a ban is a significant obstacle to their ability to operate their business sustainably. This study analyzed the potential future scenario of governments completely prohibiting e-cigarettes and found that sellers have no influence or control over this decision. They will conduct business elsewhere if the government outlaws it and they cannot contest the government’s judgments since they are final, even if it forbids them. Previous research indicated that electronic cigarettes (ECs) might pose a significant public health risk in the future for India, which is the world’s second-largest tobacco consumer with over 65% of its population under 35 years old. For now, it appears that outlawing ECs in India is the most practical course of action [56], and following example set by Punjab’s lead, other Indian states including Maharashtra, Karnataka, and Kerala also banned e-cigarettes. Additionally, countries such as Brunei, Cambodia, Indonesia, Singapore, Thailand, and Vietnam have already banned e-cigarettes [32]. Thus, resilient regulatory measures and an outright ban on e-cigarettes are required to avert future health concerns. The use of very effective marketing and promotion tactics has significantly increased the popularity of vaping and e-cigarettes. These efforts have attracted new consumers by offering innovative vaping products, causing substantial damage to the tobacco industry [57]. According to our study findings, there is a necessity of actionable laws and regulations regarding e-cigarettes in Bangladesh. In August 2016, the US Food and Drug Administration issued a formal regulation asserting its jurisdiction over e-cigarettes. This regulation established a minimum age of 18 for purchasing e-cigarettes and introduced standardized requirements, including premarket approval applications, disclosure of harmful and potentially harmful ingredients, and the use of cautionary labels [32]. All web-based sellers of e-cigarettes have to be obliged to inform customers with clear precautionary information, at the very least in the form of wording that is visibly shown on the homepage. It ought to be against the law for them to use marketing strategies, such as complimentary samples, incentive schemes, and sales campaigns, etc. [26]. Additionally, we also found out that, our experts recommended strengthening licensing requirements, imposing age restrictions on purchases, stepping up awareness campaigns about the negative health consequences of e-cigarettes, and continuous license verification for e-cigarettes selling etc., in the perspective of Bangladesh. Similar findings was found to regulate e-cigarettes, its sale is prohibited in some number of nations and in some country there need license to sale those product [58]. Many nations have implemented rules regarding the import of e-cigarettes [58].

However, one of the biggest controversies about e-cigarettes is whether these gadgets are used for medicinal purposes or as recreational substances [59]. The biggest obstacle we confront is that many young people mistakenly believe that these products are healthier substitutes for traditional, smoke-filled cigarettes. For this reason, outlawing e-cigarettes is far more important than controlling them. Therefore, enacting laws that expedite the approval process, developing robust rules about the usage of e-cigarettes, and developing future regulatory mechanisms to solve obstacles to minimize the marketing of e-cigarettes could be several good approaches regarding this issue.

### 4.1 Limitations of the study

Our study had some limitations. First of all, presenting compelling statistical data was difficult when employing the qualitative approach. Second, the interviewer occasionally felt uncomfortable responding to the questions since they were unaccustomed to delivering information using the qualitative approach, which occasionally made it difficult to determine the meaning in detail. In order to provide a universal appeal, large-scale researches that take into account the mixed-method design should be carried out to investigate this topic further.

## 5. Conclusion

This study offers a comprehensive exploration of the e-cigarette landscape in Dhaka City, Bangladesh. It uncovers significant insights into production, distribution, marketing strategies, and health perceptions. The findings undergo the urgent need for regulatory interventions and create public awareness initiatives to address the complex challenges arising from the widespread availability and marketing of e-cigarettes, particularly among the youth population. E-cigarettes are being sold in various shops where people usually go for their necessary belongings like cosmetics, watches, sunglasses, confectionary items etc. and most alarmingly surrounded by academic institutions that are expanding. Moreover, these vaping devices are being imported into Bangladesh under different codes as well as other unrecognized ways from abroad. This exposure and accessibility encourage the younger generations to consume e-cigarettes. Customers purchase e-cigarettes through online and offline means and the sellers motivate them that these are less harmful options compared to the traditional ones. The study contributes valuable knowledge for informed policymaking and public health strategies to mitigate the potential long-term consequences of e-cigarette use in the context of Dhaka City. It emphasizes the importance of a coordinated and proactive approach to safeguard public health.

## Supporting information

S1 Dataset (XLSX)

S2 Dataset (XLSX)

## Abbreviation

GIS: Geographical Information System
POS: Point-of-sale
IDI: In-depth Interview
KII: Key Informant Interview
FDA: Food and Drug Administration
TGA: Therapeutic Goods Administration
GoB: Government of Bangladesh
SDG: Sustainable Development Goal
DNCC: Dhaka North City Corporation
DSCC: Dhaka South City Corporation
GPS: Global Positioning System
IRB: Institutional Review Board
PG: Propylene Glycol
VG: Vegetable Glycerin
IC: Integrated Circuit
IQ: Intelligence Quotient
OTT: Over-the-Top
NBR: National Board of Revenue
NGO: Non-Governmental Organization
BCCP: Bangladesh Center for Communication Programs
HS: Harmonized System
EC: Electronic Cigarettes

## References

[1] Sutfin EL, McCoy TP, Morrell HER, Hoeppner BB, Wolfson M. Electronic cigarette use by college students. Drug Alcohol Depend [Internet]. 2013;131(3):214–21. Available from: doi: 10.1016/j.drugalcdep.2013.05.001 23746429 PMC3760168

[2] Liu X, Lugo A, Davoli E, Gorini G, Pacifici R, Fernández E, et al. Electronic cigarettes in Italy: a tool for harm reduction or a gateway to smoking tobacco? Tob Control. 2020;29(2):148–52. doi: 10.1136/tobaccocontrol-2018-054726 30659103

[3] Jones L. Vaping: How popular are e-cigarettes? [Internet]. BBC News. 2019 [cited 2024 Mar 28]. Available from: https://www.bbc.com/news/business-44295336

[4] Jankowski M, Krzystanek M, Zejda JE, Majek P, Lubanski J, Lawson JA, et al. E-cigarettes are more addictive than traditional cigarettes—a study in highly educated young people. Int J Environ Res Public Health. 2019;16(13):2279. doi: 10.3390/ijerph16132279 31252671 PMC6651627

[5] Heishman SJ, Kleykamp BA, Singleton EG. Meta-analysis of the acute effects of nicotine and smoking on human performance. Psychopharmacology (Berl). 2010;210:453–69. doi: 10.1007/s00213-010-1848-1 20414766 PMC3151730

[6] Shaun MMA, Nizum MWR, Shuvo MA, Fayeza F, Faruk MO, Alam MF, et al. Association between depressive symptoms and poor sleep quality among pregnant women in Northern Rural Bangladesh: a community-based cross-sectional study. BMC Psychiatry [Internet]. 2022;22(1):1–10. Available from: 10.1186/s12888-022-03839-w 35303810 PMC8933943

[7] WHO. WHO report on the global tobacco epidemic, 2023: protect people from tobacco smoke. MPOWER Packag [Internet]. 2023 [cited 2024 Mar 30];248. Available from: https://iris.who.int/bitstream/handle/10665/372043/9789240077164-eng.pdf?sequence=1

[8] Glantz SA, Bareham DW. E-cigarettes: use, effects on smoking, risks, and policy implications. Annu Rev Public Health. 2018;39:215–35. doi: 10.1146/annurev-publhealth-040617-013757 29323609 PMC6251310

[9] Jatlaoui TC. Outbreak of lung injury associated with e-cigarette product use or vaping: information for clinicians. 2019;

[10] Margham J, McAdam K, Forster M, Liu C, Wright C, Mariner D, et al. Chemical composition of aerosol from an e-cigarette: a quantitative comparison with cigarette smoke. Chem Res Toxicol. 2016;29(10):1662–78. doi: 10.1021/acs.chemrestox.6b00188 27641760

[11] Kapan A, Stefanac S, Sandner I, Haider S, Grabovac I, Dorner TE. Use of electronic cigarettes in European populations: a narrative review. Int J Environ Res Public Health. 2020;17(6):1971. doi: 10.3390/ijerph17061971 32192139 PMC7142603

[12] Wang TW. E-cigarette use among middle and high school students—United States, 2020. MMWR Morb Mortal Wkly Rep. 2020;69. doi: 10.15585/mmwr.mm6937e1 32941408 PMC7498174

[13] FDA. E-Cigarettes, Vapes, and other Electronic Nicotine Delivery Systems (ENDS) | FDA [Internet]. 2024 [cited 2024 Mar 28]. Available from: https://www.fda.gov/tobacco-products/products-ingredients-components/e-cigarettes-vapes-and-other-electronic-nicotine-delivery-systems-ends

[14] Ali FRM. E-cigarette unit sales by product and flavor type, and top-selling brands, United States, 2020–2022. MMWR Morb Mortal Wkly Rep. 2023;72. doi: 10.15585/mmwr.mm7225a1 37347717 PMC10328473

[15] Crespi E, Hardesty JJ, Nian Q, Cohen JE. Decisions of the FDA on premarket tobacco product applications: Changes in the number of unique devices and liquids used by US adults who frequently use electronic nicotine delivery systems, 2020–2023. Tob Induc Dis. 2024;22(March):1–10. doi: 10.18332/tid/184240 38482508 PMC10936557

[16] Hsu G, Sun JY, Zhu S-H. Evolution of electronic cigarette brands from 2013–2014 to 2016–2017: analysis of brand websites. J Med Internet Res. 2018;20(3):e8550. doi: 10.2196/jmir.8550 29530840 PMC5869180

[17] Dunbar MS, Martino SC, Setodji CM, Shadel WG. Exposure to the tobacco power wall increases adolescents’ willingness to use e-cigarettes in the future. Nicotine Tob Res. 2019;21(10):1429–33. doi: 10.1093/ntr/nty112 29868869 PMC6751521

[18] Blackwell AKM, De-Loyde K, Hollands GJ, Morris RW, Brocklebank LA, Maynard OM, et al. The impact on selection of non-alcoholic vs alcoholic drink availability: an online experiment. BMC Public Health. 2020;20:1–9. 32370760 10.1186/s12889-020-08633-5 PMC7201696

[19] Hollands GJ, Carter P, Anwer S, King SE, Jebb SA, Ogilvie D, et al. Altering the availability or proximity of food, alcohol, and tobacco products to change their selection and consumption. Cochrane Database Syst Rev. 2019;(9). 10.1002/14651858.CD012573.pub3 PMC6953356 31482606

[20] Willis E, Haught MJ, Morris Ii DL. Up in vapor: Exploring the health messages of e-cigarette advertisements. Health Commun. 2017;32(3):372–80. doi: 10.1080/10410236.2016.1138388 27309130

[21] Cobb NK, Brookover J, Cobb CO. Forensic analysis of online marketing for electronic nicotine delivery systems. Tob Control. 2015;24(2):128–31. doi: 10.1136/tobaccocontrol-2013-051185 24038037

[22] Grana RA, Ling PM. “Smoking revolution”: a content analysis of electronic cigarette retail websites. Am J Prev Med. 2014;46(4):395–403. doi: 10.1016/j.amepre.2013.12.010 24650842 PMC3989286

[23] Richardson A, Ganz O, Vallone D. Tobacco on the web: surveillance and characterisation of online tobacco and e-cigarette advertising. Tob Control. 2015;24(4):341–7. doi: 10.1136/tobaccocontrol-2013-051246 24532710

[24] Czogala J, Goniewicz ML, Fidelus B, Zielinska-Danch W, Travers MJ, Sobczak A. Secondhand exposure to vapors from electronic cigarettes. nicotine Tob Res. 2014;16(6):655–62. 10.1093/ntr/ntt203 PMC4565991 24336346

[25] Goniewicz ML, Kuma T, Gawron M, Knysak J, Kosmider L. Nicotine levels in electronic cigarettes. Nicotine Tob Res. 2012;15(1):158–66. doi: 10.1093/ntr/nts103 22529223

[26] Mackey TK, Miner A, Cuomo RE. Exploring the e-cigarette e-commerce marketplace: Identifying Internet e-cigarette marketing characteristics and regulatory gaps. Drug Alcohol Depend. 2015;156:97–103. doi: 10.1016/j.drugalcdep.2015.08.032 26431794

[27] GAO. Electronic Cigarettes: U.S. Imports, 2016–2018 | U.S. GAO [Internet]. 2019 [cited 2024 Mar 29]. Available from: https://www.gao.gov/products/gao-19-619r

[28] Morgan J, Jones A, Kelso C. Nicotine in electronic cigarette fluid: importation pathways to unequal harm. Intern Med J. 2021;51(7):1156–9. doi: 10.1111/imj.15412 34278688

[29] Grana R, Benowitz N, Glantz SA. E-cigarettes: a scientific review. Circulation. 2014;129(19):1972–86. doi: 10.1161/CIRCULATIONAHA.114.007667 24821826 PMC4018182

[30] Beard E, Shahab L, Cummings DM, Michie S, West R. New pharmacological agents to aid smoking cessation and tobacco harm reduction: what has been investigated, and what is in the pipeline? CNS Drugs. 2016;30(10):951–83. doi: 10.1007/s40263-016-0362-3 27421270

[31] Dyer O. India bans e-cigarettes by executive order. BMJ Br Med J. 2019;366. doi: 10.1136/bmj.l5649 31537546

[32] Barraza LF, Weidenaar KE, Cook LT, Logue AR, Halpern MT. Regulations and policies regarding e‐cigarettes. Cancer. 2017;123(16):3007–14. doi: 10.1002/cncr.30725 28440949

[33] MHFW. Smoking and Tobacco Products usage (Control) Act. Ministry of Health and Family Welfare, Government of the people’s republic of Bangladesh. 2013.

[34] Rahman MA, Joseph B, Nimmi N. Electronic Cigarettes or Vaping: Are There Any Differences in the Profiles, Use and Perceptions between a Developed and a Developing Country? Int J Environ Res Public Health. 2022;19(3). doi: 10.3390/ijerph19031673 35162695 PMC8834803

[35] Kids C for TF. Bangladesh | Tobacco Control Laws [Internet]. 2021 [cited 2024 May 5]. Available from: https://www.tobaccocontrollaws.org/legislation/bangladesh

[36] WHO. Tobacco control for sustainable development. World Health Organization, Regional Office for South-East Asia; 2017.

[37] International Conference on Civil Engineering for Sustainable Development 2014:, Hossain QS, Khulna University of Engineering & Technology BD of CE, Division I of E (Bangladesh). CE. Proceedings ICCESD 2014: 2nd International Conference on Civil Engineering for Sustainable Development, 14–16 Februrary, 2014, Khulna, Bangladesh. TA—TT -. Khulna, Bangladesh SE—xviii, 342 pages: illustrations, maps; 28 cm: ICCESD-2014, Department of Civil Engineering, Khulna University of Engineering & Technology; 2014.

[38] Ryan GW, Bernard HR. Techniques to identify themes. Field methods. 2003;15(1):85–109.

[39] Yoong SL, Hall A, Leonard A, McCrabb S, Wiggers J, Tursan d’Espaignet E, et al. Prevalence of electronic nicotine delivery systems and electronic non-nicotine delivery systems in children and adolescents: a systematic review and meta-analysis. Lancet Public Heal. 2021;6(9):e661–73. doi: 10.1016/S2468-2667(21)00106-7 34274048 PMC8390387

[40] Christensen T, Welsh E, Faseru B. Profile of e-cigarette use and its relationship with cigarette quit attempts and abstinence in Kansas adults. Prev Med (Baltim). 2014;69:90–4. doi: 10.1016/j.ypmed.2014.09.005 25230365

[41] Simmons VN, Quinn GP, Harrell PT, Meltzer LR, Correa JB, Unrod M, et al. E-cigarette use in adults: a qualitative study of users’ perceptions and future use intentions. Addict Res Theory. 2016;24(4):313–21. doi: 10.3109/16066359.2016.1139700 27725794 PMC5055066

[42] Banks E, Yazidjoglou A, Joshy G. Electronic cigarettes and health outcomes: epidemiological and public health challenges. Int J Epidemiol. 2023;52(4):984–92. doi: 10.1093/ije/dyad059 37192053 PMC10396413

[43] Meo SA, Al Asiri SA. Effects of electronic cigarette smoking on human health. Eur Rev Med Pharmacol Sci. 2014;18(21):3315–9. 25487945

[44] Kim JY, Kang HS, Jung JW, Jung SY, Park HJ, Park JS, et al. Nicotine dependence and stress susceptibility in E-cigarette smokers: The Korea national health and nutrition examination survey 2013–2017. Tuberc Respir Dis (Seoul). 2021;84(2):159–66. doi: 10.4046/trd.2020.0166 33401344 PMC8010419

[45] Hanafin J, Sunday S, Clancy L. Friends and family matter Most: a trend analysis of increasing e-cigarette use among Irish teenagers and socio-demographic, personal, peer and familial associations. BMC Public Health. 2021;21(1):1–12. 34732172 10.1186/s12889-021-12113-9 PMC8567623

[46] Glasser A, Abudayyeh H, Cantrell J, Niaura R. Patterns of e-cigarette use among youth and young adults: review of the impact of e-cigarettes on cigarette smoking. Nicotine Tob Res. 2019;21(10):1320–30. doi: 10.1093/ntr/nty103 29788314

[47] Bhalerao A, Sivandzade F, Archie SR, Cucullo L. Public health policies on e-cigarettes. Curr Cardiol Rep. 2019;21:1–6. 31463564 10.1007/s11886-019-1204-y PMC6713696

[48] Vedøy TF, Lund KE. Self-reported sources for distribution of cigarettes, snus and e-cigarettes. Tidsskr den Nor laegeforening Tidsskr Prakt Med ny raekke. 2017;137(16). 10.4045/tidsskr.16.0994 28871760

[49] Jensen JLK, Rebentisch K, Tripp HL, Merten JW. Price, convenience, the buying experience, and other motivations for purchasing tobacco and e-cigarettes online. Tob Induc Dis. 2022;20. doi: 10.18332/tid/152138 36118561 PMC9437897

[50] Chankaew T, Baiya P, Chinwong D, Yoodee V, Chinwong S. Electronic cigarettes in Thailand: behaviour, rationale, satisfaction, and sex differences. Int J Environ Res Public Health. 2022;19(14):8229. doi: 10.3390/ijerph19148229 35886084 PMC9323309

[51] Trade Commission F. Federal Trade Commission Cigarette Report for 2014. 2015;31. Available from: https://www.ftc.gov/system/files/documents/reports/federal-trade-commission-cigarette-report-2014-federal-trade-commission-smokeless-tobacco-report/ftc_cigarette_report_2014.pdf

[52] Struik LL, Dow-Fleisner S, Belliveau M, Thompson D, Janke R. Tactics for drawing youth to vaping: Content analysis of electronic cigarette advertisements. J Med Internet Res. 2020;22(8):1–11. doi: 10.2196/18943 32663163 PMC7455879

[53] Scaioli G, Bert F, Martorana M, Gili R, Thomas R, Gualano MR, et al. Advertisement of electronic cigarettes in Italy: characteristics of online videos and the most popular promotional messages. Health Educ Res. 2018;33(6):473–80. doi: 10.1093/her/cyy030 30247572

[54] Kornfield R, Huang J, Vera L, Emery SL. Rapidly increasing promotional expenditures for e-cigarettes. Tob Control. 2015;24(2):110–1. doi: 10.1136/tobaccocontrol-2014-051580 24789603 PMC4214902

[55] Paradise J. Electronic cigarettes: smoke-free laws, sale restrictions, and the public health. Am J Public Health. 2014;104(6):e17–8. doi: 10.2105/AJPH.2014.301890 24825224 PMC4062010

[56] Kaur J, Rinkoo AV. A call for an urgent ban on e-cigarettes in India—a race against time. Glob Health Promot. 2015;22(2):71–4. doi: 10.1177/1757975914537322 24938513

[57] Tanski SE. Marketing and advertising of e-cigarettes and pathways to prevention. Electron Cigarettes Vape Devices A Compr Guid Clin Heal Prof. 2021;105–14.

[58] Kennedy RD, Awopegba A, De León E, Cohen JE. Global approaches to regulating electronic cigarettes. Tob Control. 2017;26(4):440–5. doi: 10.1136/tobaccocontrol-2016-053179 27903958 PMC5520254

[59] Abbasi J. FDA extends authority to e-cigarettes: implications for smoking cessation? Jama. 2016;316(6):572–4. doi: 10.1001/jama.2016.8568 27419929

